# Association between oral and fecal microbiome dysbiosis and treatment complications in pediatric patients undergoing allogeneic hematopoietic stem cell transplantation

**DOI:** 10.1038/s41598-024-55690-6

**Published:** 2024-03-20

**Authors:** M. Faraci, C. Bonaretti, G. Dell’Orso, F. Pierri, S. Giardino, F. Angiero, S. Blasi, G. Farronato, E. Di Marco, A. Trevisiol, E. Olcese, L. Rufino, M. Squillario, R. Biassoni

**Affiliations:** 1 Hematopoietic Stem Cell Transplant Unit, Department of Hemato-Oncology, IRCCS Istituto Giannina Gaslini, Genova, Italy; 2 Molecular Diagnostic Laboratory, IRCCS Istituto Giannina. Gaslini, Genova, Italy; 3 Department of Surgical and Diagnostic Sciences, University of Genova, Genova, Italy; 4 Department of Biomedical, Surgical and Dental Sciences, University of Milan, Milano, Italy; 5 Laboratory of Clinical Analysis, IRCCS Istituto G. Gaslini, Genova, Italy; 6 IRCCS Ospedale Policlinico San Martino, Genova, Italy

**Keywords:** Oral microbiome, Gut microbiome, Hematopoietic stem cell transplant (HSCT), Mucositis, Graft versus host disease (G*v*HD), Microbiology, Oncology, Paediatric research

## Abstract

The oral and gastrointestinal mucosae represent the main targets of the toxic effect of chemo and/or radiotherapy administered during the conditioning regimen before hematopoietic stem cell transplant (HSCT). These harmful consequences and the immunological complications that may occur after the transplant (such as Graft versus Host Disease, G*v*HD) are responsible for the clinical symptoms associated with mucositis during the aplasia phase, like pain, nausea, vomiting, and diarrhea. These toxicities could play a critical role in the oral and gastrointestinal microbiomes during the post-transplant phase, and the degree of microbial dysbiosis and dysregulation among different bacterial species could also be crucial in intestinal mucosa homeostasis, altering the host’s innate and adaptive immune responses and favoring abnormal immune responses responsible for the occurrence of G*v*HD. This prospective pediatric study aims to analyze longitudinally oral and gut microbiomes in 17 pediatric patients who received allogeneic HSCT for malignant and non-malignant diseases. The oral mucositis was mainly associated with an increased relative abundance of *Fusobacteria*, and *Prevotella* species, while *Streptococcus descendants* showed a negative correlation. The fecal microbiome of subjects affected by cutaneous acute G*v*HD (aG*v*HD) correlated with *Proteobacteria*. Oral mucosal microbiota undergoes changes after HSCT, *Fusobacteria,* and *Prevotella* represent bacterial species associated with mucositis and they could be the target for future therapeutic approaches, while fecal microbiome in patients with acute G*v*HD (aG*v*HD) revealed an increase of different class of *Proteobacteria* (*Alphaproteobacteria* and *Deltaproteobacteria*) and a negative correlation with the class of *Gammaproteobacteria*.

## Introduction

The oral bacterial ecosystem represents a complex equilibrium frequently challenged by habits such as poor oral hygiene as well as the use of antimicrobial agents, physiological hormonal changes, age and dental development, and external pathogens. When this balance is lost, dysbiosis occurs with pathological complications^1^. The microbiota plays a crucial role in regulating host-microbe interactions among bacterial species, and its sensitivity to medical treatment can influence inflammation and the breakdown of the mucosal barrier^2,3^. In the context of hematopoietic stem cell transplantation (HSCT), both the oral and gut microbiota can become dysbiotic due to various factors that can affect the intestinal ecosystem and cause damage to both the oral and intestinal mucosa. These factors can include the use of antibiotics, chemotherapeutic drugs, alloimmune reactions like GvHD, and prolonged inflammatory states^4,5^. Oral mucositis (OM) represents one of the most frequent clinical complications observed in patients who received chemotherapy for the treatment of malignant diseases or during the conditioning regimen (CR) before HSCT^6–8^. Myeloablative regimens (MAC) are more frequently associated with severe toxic complications, including oral and gastro-intestinal mucositis, compared to reduced intensity conditioning regimens (RIC), and it is recognized that the development of severe mucositis is correlated with the performance status at transplant and with the previous toxicity occurred before the HSCT^3^. Patients with OM could develop oral ulceration associated with intense pain that compromises oral intake, and even lead to bloodstream infections, resulting in a decreased patient’s quality of life, prolonged hospitalization, and high treatment costs. The occurrence of infection starting from the oral cavity is facilitated by the post-transplant neutropenia phase^9^. The increased possibility of the risk of infection in transplanted patients is related to the immunosuppressive therapy administered to prevent and treat G*v*HD that represents “per se” the higher cause of immunodepression. The relationship between acute toxicity and the occurrence of G*v*HD is known, and who received MAC have a higher risk to develop acute toxicity compared to those who received RIC. Moreover, gastrointestinal G*v*HD induces a loss of intestinal flora diversity and alteration of microbiota^10^.

This study aims to analyze the oral and intestinal microbiome in a cohort of pediatric patients who received allogeneic HSCT and to describe the microbiological characteristic of their microbiome in the different phases of transplant and in patients who developed oral/gastrointestinal (GI) mucositis and acute G*v*HD.

## Methods

### Study design

For this prospective single-center study, we included 17 patients who received an allogeneic HSCT at IRCCS Istituto G. Gaslini from 2019 to 2022. The following variables: gender, age, underlying disease, oral and GI mucositis and its grade, type of CR, G*v*HD prophylaxis, and acute and chronic G*v*HD were included in the analysis. A prospective daily patient oral assessment was recorded, including diet, pain, erythema, ulceration, and the oral mucositis grade based on the World Health Organization (WHO) grading scale. Patient with acute gastrointestinal toxicity was recorded using Common Terminology Criteria of Adverse Events (CTCAE) grading. Acute and chronic G*v*HD was assessed using the standard criteria^11,12^. Samples for oral and fecal microbiome analysis were collected before HSCT, at engraftment, and 30 or 100 days after transplant. All patients gave their informed consent to participate in the study.

The study was conducted following the Declaration of Helsinki, and the protocol was approved by the Ethics Committee of Regione Liguria 369/2018.

### Sample collection

The specimens were sampled in the morning without the patient having drunk, or eaten anything, performed oral hygiene in the previous half hour, or used mouthwashes or oral antiseptics, which would affect the result of the microbiological analyses. The swab was placed at the level of the mouth vestibule of the lower arch and rubbed about ten times along the entire length of each gingival semi-arch. At the end of the collection, the swab was repositioned inside the tube containing a stabilizing solution and vigorously shaken 15 times. Stools samples were collected and immediately transferred into a Omnigene-Gut tube, (DNA Genotek, 3000–500 Palladium Drive, Ottawa, ON, Canada, K2V 1C2) for stabilization of microbial DNA from feces for gut microbiome profiling. In the end, the sample was transferred jointly with the fecal specimen to the acceptance of the laboratory. Biological materials and extracted DNA were conserved at − 20 °C before any additional processing and kept at − 20 °C for a short period until library preparation and sequencing.

### DNA extraction

DNA extraction from fecal samples was performed by resuspending into 1 ml of the ASL Stool lysis buffer (Qiagen GmbH, Germany) a tiny quantity of fecal material by vigorous pipetting. Instead, oral swab samples were resuspended in a 200 µl physiologic solution. Then the resuspended materials were processed to extract DNA using the MagDEA DNA 200GC extraction kit and PSS Magtration System 12GC automated platform, according to the manufacturer’s instructions (Precision System Science PSS Co., Ltd, Matsudo, Japan). DNA was eluted into 100 µl of 10 mM TE buffer; quality and quantity were evaluated by spectrophotometric and Qubit fluorimetric quantitation assays, respectively (ThermoFisher Scientific, Waltham, MA).

### 16S rRNA sequencing and data analysis

In detail, 3 ng of DNA was used for each 16S amplification reaction performed with Ion 16S Metagenomics Kit™ (ThermoFisher Scientific, Waltham, MA). It allows the analysis by PCR amplification of 7 out of 9 informative 16S polymorphic regions (V2, V4, V8, V3, V6-7, V9) according to the manufacturer protocols. IonPlus-Library kit for AB library builder (ThermoFisher Scientific, Waltham, MA) was used for library synthesis. Different bar-coded libraries were automatically handled and loaded onto an Ion 520 chip by the Ion Chef System and then sequenced by the GeneStudio S5 system (ThermoFisher Scientific, Waltham, MA).

Data analysis was performed with Ion Reporter ™ suite software (v 5.18) using both the curated Greengenes (v13.5) and the premium curated MicroSEQ ID 16S rRNA reference library (v2013.1) databases with standard parameters.

### Bioinformatics and statistics

Compositional/functional profiling and the comparative analysis of microbiome data were performed with MicrobiomeAnalyst^13–15^. Data filtering for low abundance and low variance OTUs (based on the prevalence in 20% of samples and interquartile range or iqr set at 10%) was applied for all the relative abundance comparisons using different algorithms (metagenomeSeq, EdgeR, DESeq2, LDA, and LEfSe). All p-values have been adjusted to correct for multiple hypotheses, using Benjamini and Hochberg false discovery rate (FDR < 0.05), unless differently specified. In the comparison of cases and the controls, the SparCC (Sparse Correlations for Compositional Data) analysis was used^16^. SparCC is a computational tool that analyses the interactions between different microbial species in the microbiome. SparCC uses a technique called "sparse inverse covariance estimation" to identify patterns of interactions in the data and to infer the underlying network of interactions between the components. SparCC is often used to identify keystone species, which may participate in maintaining the overall eubiosis. MD-index (MDI) is the natural logarithm of the ratio of the sum of the abundance of all the taxa that are enriched, and the ones depleted^17^. The microbial dysbiosis index measures the imbalance or deviation of the microbial community from a healthy or normal state. The MDI is calculated using the relative abundance of different microbial taxa and comparing it to a reference or control population (in our case situation before transplant). The value of the number higher or lower than zero indicates a greater degree of dysbiosis or abnormal microbial composition, while a value around zero denoted a normal or healthy microbial population.

### Ethical approval

Informed consent was obtained from all individual participant, or their family included in the study. The studies involving human participant were conducted in accordance with the Declaration of Helsinki and were reviewed and approved by the Ethics Committee of Regione Liguria 369/2018 issued for IRCCS Istituto Giannina Gaslini—Genova, PI Dr Maura Faraci.

## Results

### Patients

During the period between 2019 and 2022, 17 patients (median age 6.0 years, range 0.7–24.7 years) who underwent allogeneic HSCT were enrolled in the study, 9 of them affected by non-malignant diseases (3 bone marrow failures, 5 congenital immunodeficiencies, and one autoinflammatory disease) and 8 by malignant diseases (5 acute lymphoblastic leukemia and 3 acute myeloid leukemia) (Table 1). 8 patients received stem cells from a haploidentical donor, 5 from an alternative donor, and 4 from a related donor. A myeloablative (MAC) CR was used in 16 patients, including total body irradiation (TBI) in 5 patients, busulfan (Bus) in 3 patients, and treosulfan (Treo) in the remaining 8 patients. One patient received a reduced-intensity CR (RIC) for congenital dyskeratosis. Out of 17 patients enrolled in the study, 15 patients with age ≥ 2 years and with a primary or secondary dentition were included in the statistical analysis. In addition, the same cut-off was valid for estimating microbiome composition^18^.

**Table 1:** Characteristics of the transplanted patients.

Code	Age (yrs)	Underlying Disease	Type of donor	Conditioning regiemn	O-M	GI-M	Infections	Acute GVHD	Chronic GVHD
1	2.9	CGD	UD	Treosulfan-FludarabineCampath	2	0	0	2	0
2	18.8	CGD	RD	Treosulfan-Fludarabine- ATG	1	0	0	0	0
3	2.4	CGD	UD	Treosulfan-FludarabineCampath	0	0	0	2	0
4	2.6	TACI-CARD11 deficiency	Haploidentical α/β/CD19 depletion	Treosulfan-Fludarabine-T*hiotepa*	0	2	0	0	0
5	10.9	Hyporegenerative anemia	RD	Treosulfan-Fludarabine- ATG	2	2	0	3	0
6	14.1	GATA-2insufficiency	Haploidentical α/β/CD19 depletion	Treosulfan-Fludarabine-*hiotepa*	1	0	0	1-Gastric	0
7	24.7	Bone Marrow aplasia	UD	TreosulfanFludarabine-*Thiotepa*	0	0	0	0	0
8	2	Dyskeratosis congenita 2ndHSCT	Haploidentical α/β/CD19 depletion	Fludarabine-*Cyclophosphamide* Campath	1	0	HCMV	0	0
9	4.2	ALL	Haploidentical CY- post	TBI-Fludarabine-CY -post	1	0	0	0	0
10	17.3	AML	UD	TBI-Fludarabine-L-PAM	4	0	0	3	1
11	13.9	AML	RD	Busulfan Fludarabine-L-PAM	4	0	HCMV	0	0
12	11.2	ALL	UD	TBI-Etoposide	3	0	pneumonia	2	0
13	6.0	ALL	Haploidentical CY-post	TBI-FludarabineEX-post	0	0	0	0	0
14	13.9	ALL	Haploidentical CY-post	TBIFludarabine-EX-post	1	0	*Stenotrophomonas*	2	0
15	2.0	AML	Haploidentical CY-post	Busulfan –Thiotepa-Fludarabine Cy-post	0	0	0	3-Gut	0
16	0.7	Mevalonic aciduria	Haploidentical α/β/CD19 depletion	Treosulfan –Thiotepa-Fludarabine	0	0	0	0	0
17	0.7	ALL	RD	Busulfan –Thiotepa Fludarabine	2	0	0	3-Gut	0

CGD: Chronic granulomatosis disease; ALL: Acute lymphoblastic leukemia: AML: Acute myeloid leukemia) O-M (Oral mucositis), GI-M (Gastro-Intestinal Mucositis), Campath (alemtuzumab), ATG (anti-thymocyteglobulin), RD (Related Donor), UD (Unrelated Donor), HCMV (Human CytoMegaloVirus), TBI (Total Body Irradiation), L-PAM (Melphalan), CY post (cyclophosphamide post tranplant); G*v*HD = Graft versus Host Disease. Acute or Chronic GVHD (grade-localization, if any).

In detail, 11 patients developed OM subdivided into grade 1 (n = 5), grade 2 (n = 3), grade 3 (n = 1), and grade 4 (n = 2). 7 patients who received HSCT for non-malignant developed mild OM (grade 0 in 4, grade 1 in 3, and grade 2 in 2), while patients transplanted for the malignant disease had more severe OM (grade 0 in 2, grade 1 in 2, grade 2 in 1, grade 3 in 1, grade 4 in 2). GI-mucositis was observed only in 2 patients (grade 2) who received HSCT for non-malignant disease. Acute G*v*HD (aG*v*HD) was observed in 8 patients, 4 of them had cutaneous acute G*v*HD above grade 2, and 3 with a GI aG*v*HD (grade 3). Limited chronic G*v*HD was present in one patient (Table 1).

Regarding nutrition, it is known that there are different recommendations for the use of enteral or parenteral feeding^19^. In our case, 15 patients received total parenteral nutrition for a median of 26.8 days (12–55 days).

### Alpha and beta diversity indexes

**Figure 1:**
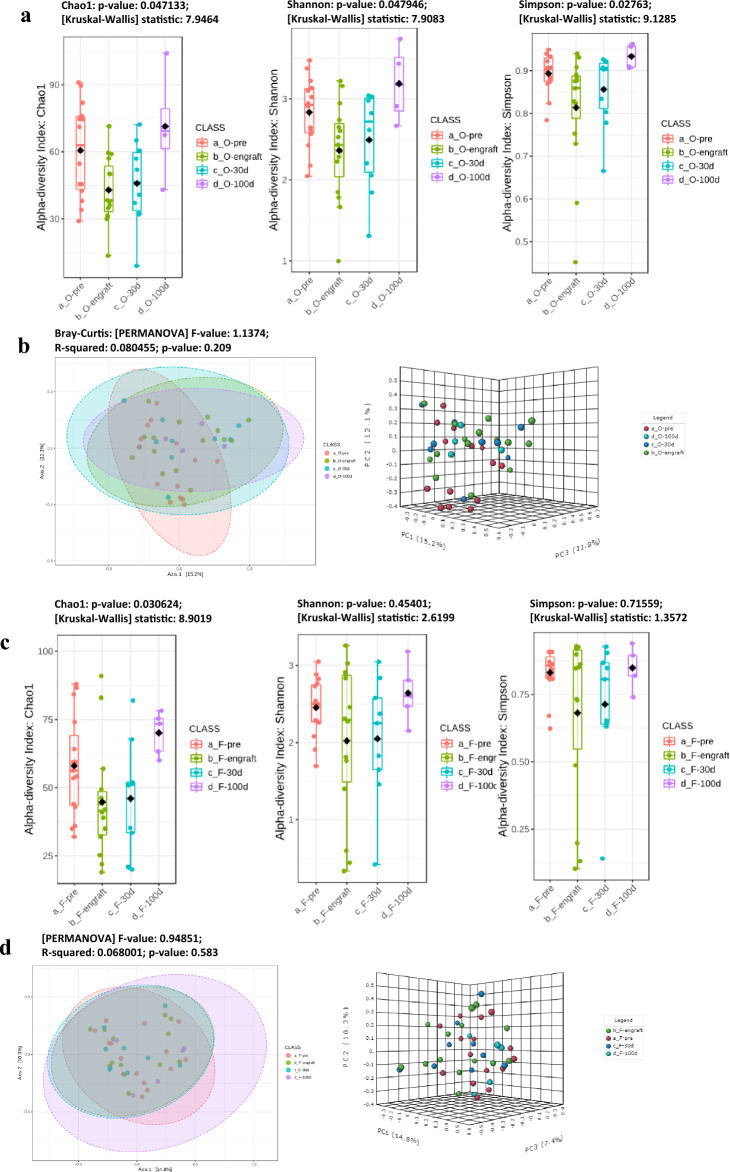
Alpha diversity results comparing the complete cohort of oral (**a**) and fecal (**c**) microbiomes of patients > 2 years old. From the left, the alpha-indexes estimate the community richness (Chao1-index) or community richness and evenness (Shannon or Simpson indexes). The beta diversity Bray–Curtis’s distance matrix analysis for oral (**b**) and fecal (**d**) samples. The comparison performed with the Principal Coordinates Analysis (PCoA) showed using a 2D or 3D distribution. Permutational Multivariate Analysis of Variance (PERMANOVA) was used for statistical significance analysis.

The alpha diversity profiling indexes used to analyze the microbial community in oral samples were the Chao1, Shannon, or Simpson indexes. The first index provided evidence of a correlation among microbiome richness, while the Shannon and Simpson indexes provided information about community richness and evenness. Together, these metrics offer valuable insight into the structure and composition of the microbiome. All the indexes showed in the oral swabs the lowest values at the HSCT engraftment, while they increased gradually with the highest values after 100 days. All alpha indexes analyzed reached statistical significance in oral swab samples (Fig. 1a), but only the Chao1 reached significance in fecal samples (Fig. 1c).

According to the data, it appears that the samples taken before transplant from patients affected by malignant diseases, both in oral and stool, had lower values in all three alpha diversity indexes as compared to the samples from patients with non-malignant diseases. Based on the analysis performed at engraftment, it seems that there was a similar trend observed in the samples taken from patients affected by malignant diseases, with a single exception. The Chao1 index of oral microbiome resulted in higher values for samples from malignant diseases (seven patients, Chao1: 26.07 + / − 7.52 on) as compared to non-malignant diseases (seven patients, Chao1: 20.14 + / − 7.08).

Beta diversity was analyzed using the Bray–Curtis index and PERMANOVA statistical evaluation without reaching significance either in oral or fecal samples (Fig. 1b, d).

### Oral and fecal microbiome analysis and taxonomic determination by 16S-targeted NGS sequencing

We analyzed 98 oral and fecal microbiomes that gave 194,521 ± 57,929 16S mapped reads per sample. These reads identified 1524 ± 551 operational taxonomic units (OTUs) in the 49 oral swab samples and 1545 ± 389 OTUs in the fecal samples analyzed. The analysis of oral microbiomes indicated that the abundance of the Firmicutes phylum increased over the time points studied (pre-HSCT, at engraftment, + 30 days, and + 100 days after HSCT), rising from 46 to 72%. However, the Proteobacteria and Bacteroidetes phyla showed the opposite trend, with a decrease in abundance from 20 to 10% and 17% to 9%, respectively. Accordingly, the analysis of *Firmicutes/Bacteroidetes* (F/B) or *Firmicutes/Proteobacteri*a (F/P) ratios showed that both increased their value along the four observations, with F/B ranging from 2.7 to 8.0 and F/P from 2.3 to 7.2 (Table 2a). The abundance of Fusobacteria halves after 100 days from transplant only, according to Table 2a. On the other hand, the fecal microbiomes showed a different behavior, with *Firmicutes* maintaining a stable abundance rate, *Bacteroidetes* increasing, and *Proteobacteria* decreasing their fullness along longitudinal points, as shown in Table 2b.

**Table 2:** Longitudinal analysis of relative abundance (%) of major phyla, and analysis of F/B or F/P ratios indicative of dysbiosis.

Phyla	a: oral all samples				b: fecal all samples			
	pre-HSCT^14^	Engraftment^15^	HSCT + 30d^9^	HSCT + 100d^5^	pre-HSCT^15^	Engraftment^14^	HSCT + 30d^9^	HSCT + 100d^5^
*Actinobacteria*	11	9	7	7	3	4	1	3
*Bacteroidetes*	17	14	11	9	22	19	19	33
*Firmicutes*	46	55	60	72	43	44	50	44
*Fusobacteria*	6	6	5	3	1	2	0	0
*Proteobacteria*	20	16	17	10	31	31	29	20
F/B	2,7	3,9	5,5	8,0	2,0	2,3	2,6	1,3
F/P	2,3	3,4	3,5	7,2	1,4	1,4	1,7	2,2

Analysis was performed in all samples from oral or fecal microbiome, analyzing samples from patients with malignant or non-malignant pathologies. F/B *Firmicutes/Bacteroidetes* ratio, F/P *Firmicutes/Proteobacteria* ratio, In squared parenthesis were indicated the number of samples for each time point.

**Figure 2:**
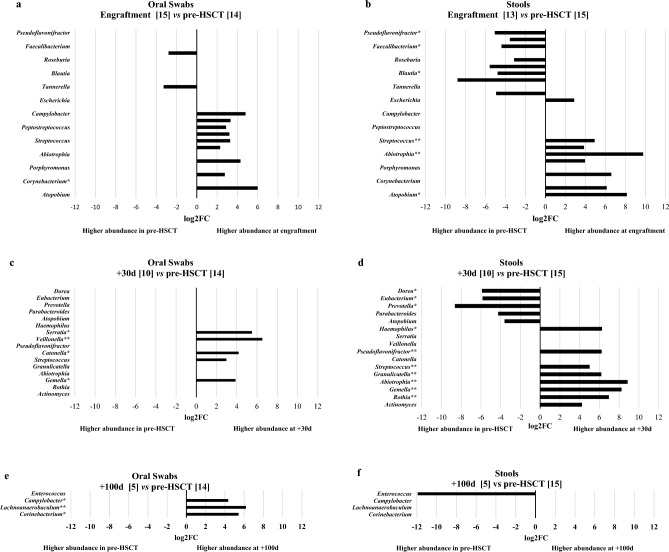
Longitudinal analysis using the EdgeR method for differential expression analyses was conducted to identify the difference in abundance of various taxa between oral (**a**, **c**, **e**) and stool (**b**, **d**, **f**) samples at different time points always compared with the microbiome of samples before HSCT transplant. All the analyses were focused on the genus level. The numbers in squared parenthesis represent the number of patients belonging to the indicated group of samples. The fold change value is represented logarithmically on base 2 (log2FC), and it shows the increase or decrease of abundance of a particular taxa in the comparison between the two groups of samples. FDR (False Discovery Rate) indicates the statistical significance value after adjustment for multiple comparisons. All statistical analyses showed FDR < 0.05, * < 0.01, or ** < 0.001.

The findings of the analysis of taxa abundances in oral or fecal microbiomes at engraftment compared to the situation before HSCT suggest that there were significant changes in the abundance of some genera in both oral and fecal samples (Fig. 2a and b, Supplementary material Table S1). For instance, the genera *Abiotrophia* and *Streptococcus* were more significantly abundant at engraftment in both oral and fecal samples. It’s interesting to note that some genera such as *Atopobium*, *Rothia*, *Actinomyces*, *Gemella*, *Granulicatella*, and *Escherichia* were significantly more abundant in the fecal microbiome at engraftment, while the *Corynebacterium*, *Serratia*, *Campylobacter*, *Catonella*, *Peptostreptococcus*, *Veillonella*, and *Porphyromonas* were found to be more abundant at engraftment in oral microbiomes. Additionally, the findings showed that *Prevotella*, *Dorea*, *Pseudoflavonifractor*, *Bifidobacterium*, *Blautia*, *Faecalibacterium*, *Eubacterium*, and *Roseburia* were significantly more abundant before HSCT in fecal samples only, and the same for *Tannerella*, and *Stomatobaculum* was in oral microbiomes. After 30 days following transplant, the genera *Gemella* and *Streptococcus* were found to be significantly more abundant in both oral and fecal samples as shown in Fig. 2c and d, respectively (Supplementary material Table S1). At 30 days after HSCT, *Abiotrophia* and *Rothia* were the genera with the highest value of abundance increase in fecal microbiomes. On the other hand, in oral microbiomes, *Veillonella* and *Serratia* were found to be more abundant at + 30 days. Finally, the genera typically more abundant in samples before HSCT were *Prevotella* and *Dorea* in fecal microbiome comparisons (Fig. 2).

### Microbiome associated with oral mucositis

**Figure 3:**
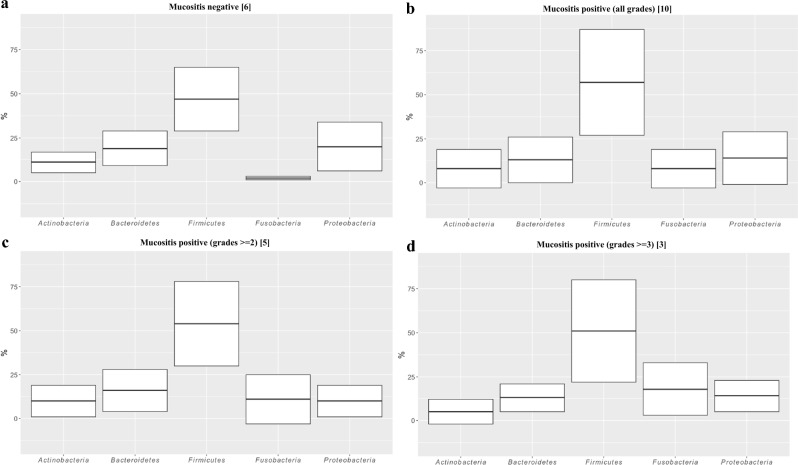
Phila abundances in oral swabs at engraftment in patients treated with myeloablative conditioning (MAC). Microbiome from Oral-mucositis (OM) negative samples (**a**) compared to OM positive (all grades) specimens (**b**), OM positive grades >  = 2 (**c**), or affected by severe mucositis (OM grades >  = 3) in panel. (**d**) In square brackets, it was indicated the number of patients belonging to the groups in the analysis. A horizontal line splitting each box in two indicates the median. The box limit indicates the upper and lower quartiles of the data.

**Figure 4:**
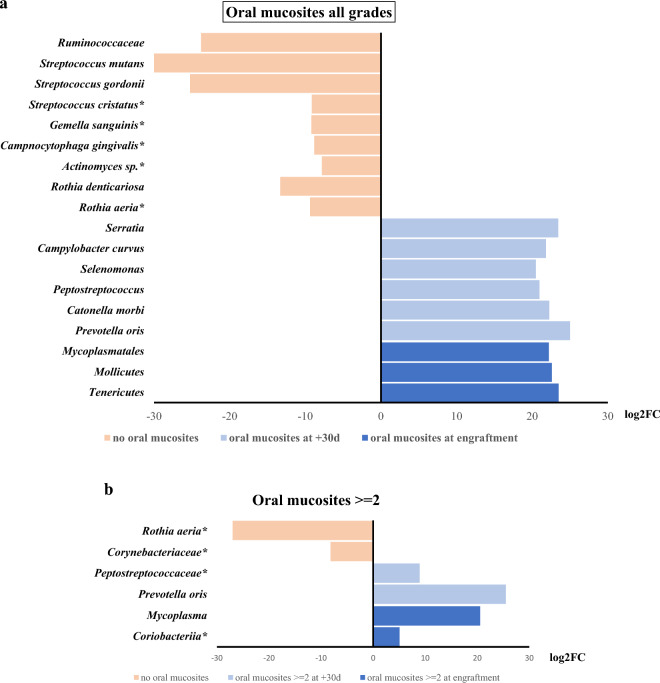
(**a**) The figure showed the DESeq2 analysis to identify the difference in abundance of various taxa between oral samples that developed oral mucositis of all grades, either at engraftment (10 patients) or (**b**) after 30 days from it (7 patients) and specimens that never had mucositis (5 patients). The fold change value, represented as logarithmic on base 2 (log2FC), shows the increase or decrease of abundance of a particular taxa in the comparison between the two groups of samples. FDR (False Discovery Rate) indicates the statistical significance value after adjustment for multiple comparisons. All statistical analyses showed FDR < 0.001, except for the taxa indicated with an asterisk (*) that had FDR < 0.05.

Oral mucositis (OM) is a common complication associated with hematopoietic stem cell transplantation (HSCT). OM refers to the inflammation and ulceration of the mucous membranes lining the mouth, throat, and gastrointestinal tract. Occurs because of the high-dose chemotherapy and/or radiation therapy that patients undergo as part of the transplant conditioning regimen. The severity of oral mucositis can vary, ranging from mild discomfort to severe pain, and it can significantly impact a patient’s quality of life. It’s important to note that both malignant and non-malignant disease patients experienced complications such as OM. This emphasizes the importance of implementing personalized care and treatment plans that are specifically designed to address the unique health needs of each patient. Furthermore, the analysis of phyla-abundances during engraftment revealed a strong positive correlation between the increasing expansion of *Fusobacteria* and the severity of oral mucositis, highlighting the need for further research into the underlying mechanisms driving this relationship. It’s interesting to note that patients who did not exhibit OM had only 2% of *Fusobacteria* in their samples (Fig. 3). According to the data, the abundance of *Fusobacteria* increased from 8 to 18% in OM-positive specimens, with the highest percentage found in more severe cases of mucositis (grade ≥ 3), as shown in Fig. 3. It’s interesting to note that a longitudinal analysis of differential abundances in oral samples with mucositis (all grades) showed that *Mycoplasmatales* descendants and the *Prevotella oris* species were among the taxa with the most statistically significant increase in specimens at engraftment or after 30 days from HSCT, respectively. (Fig. 4a, Supplementary material Table S4). Moreover, in the oral microbiome, *Mycoplasma* (at engraftment) and *Prevotella oris* (after 30 days from transplant) were also associated with the best statistical correlation with grade ≥ 2 OM in oral swab samples (Fig. 4b, Supplementary material Table S5). On the contrary, *Ruminococcaceae*, *Rothia*, and the *Streptococcus* genus in oral swab samples were found relevant, from a statistical point of view, in patients that never reported oral mucositis, so to be considered as protective taxa (Figs. 4a, 3b, Supplementary material Tables S4, and S5).

On the contrary, the analysis of fecal microbiome showed *Egghertella lenta*, *Bacteroides ovatus*, *Ruminococcus gnavus*, *Lachnoclostridium lavalense*, *Pseudoflavonifractor*, and different species of *Clostridium* always associated with specimens of reporting oral mucositis (Fig. 4, Supplementary material Tables S4, and S5).

### Networks of interaction among microbial taxa in oral mucositis

**Figure 5:**
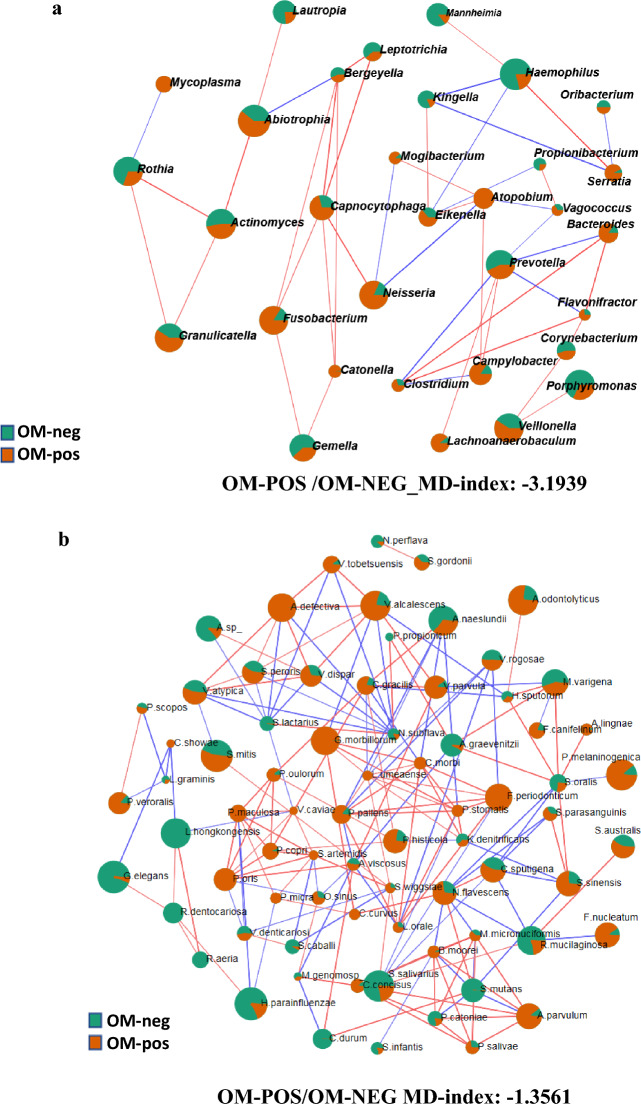
(**a**) SparCC analysis of oral microbiome at engraftment comparing samples with mucositis (of any grade) to ones negative for such complication. All patients underwent a myeloablative conditioning regimens (MAC). Each node represents genera that were differently colored in consideration of the preferential abundance in mucositis-positive (orange) or mucositis-negative samples (green). Red lines connecting nodes indicate a positive correlation between taxa, while blue lines showed a negative one. MD-index represents the dysbiosis-index of cases over controls. Network was computed for *p* <  = 0.05 and correlation threshold was set at 0.3. (**b**) Oral microbiome after 30 days from transplant among samples with severe oral mucositis (grades >  = 2) compared to samples negative for such complication. Each node represents a species differently colored in consideration of the preferential abundance in mucositis positive (orange) or mucositis negative samples (green). Red lines connecting nodes indicate a positive correlation between taxa, while blue lines show a negative one. MD-index represents the dysbiosis-index of cases over controls. Network was computed for *p* <  = 0.05 and the correlation threshold was set at 0.5.

The network correlation between the taxa abundance and OM was also studied in the oral microbiome using sparse correlation for compositional data (SparCC) analysis. The MD-Index for each informative comparison was also calculated. Thus, in patients undergoing the myeloablative (MAC) regimen, the MD-Index at engraftment of cases of patients developing mucositis of any complexity, compared to samples from patients that never developed mucositis analyzed at the level of genera was − 3.1939 (Fig. 5a) and − 1.5818, the one associated with mucositis with more complexity (grades ≥ 2). These data indicated that an increased MD-Index value correlates with more complex mucositis. The SparCC analysis of the microbiome associated with OM of any grade indicated that 29 genera produce positive/negative correlations (threshold 0.3, *p* ≤ 0.05). Among these genera, two third (at engraftment) and approximately three-quarters of 30 genera (after 30 days from HSCT) showed positive correlations among them. Overall, the *Prevotella* genus showed the highest number of correlations with other taxa (seven), computed by the SparCC network analysis (Fig. 5a). The *Capnocytophaga* showed six correlations all only with positive sign. Interestingly, the species *Atopobium parvulum* and *Capnocytophaga sputigena* confirmed their association with mucositis (Fig. 5b), as already suggested in patients treated with radiotherapy for haematologic malignancies^20,21^. More, the analysis of the microbial species associated with OM (grades ≥ 2) in patients subjected to MAC regimen showed a network of 178 species (threshold of 0.3 and a *p* ≤ 0.05), 106 of them producing positive correlations and the MD-Index of cases over controls after 30 days from transplant was 0.6979 (Fig. 5b). *Streptococcus*, *Prevotella*, and *Veillonella* were among the genera showing the highest correlation numbers with other taxa in the network analysis after 30 days from transplant. In addition, 12, 10, and 8 were the species found to belong to these genera. Thus, indicating their importance in the association with OM. Overall, these network analyses on oral microbiome pinpointed that *Prevotella* correlates positively with OM samples, while *Streptococcus* was always associated with a negative correlation.

### Acute GVHD

**Figure 6:**
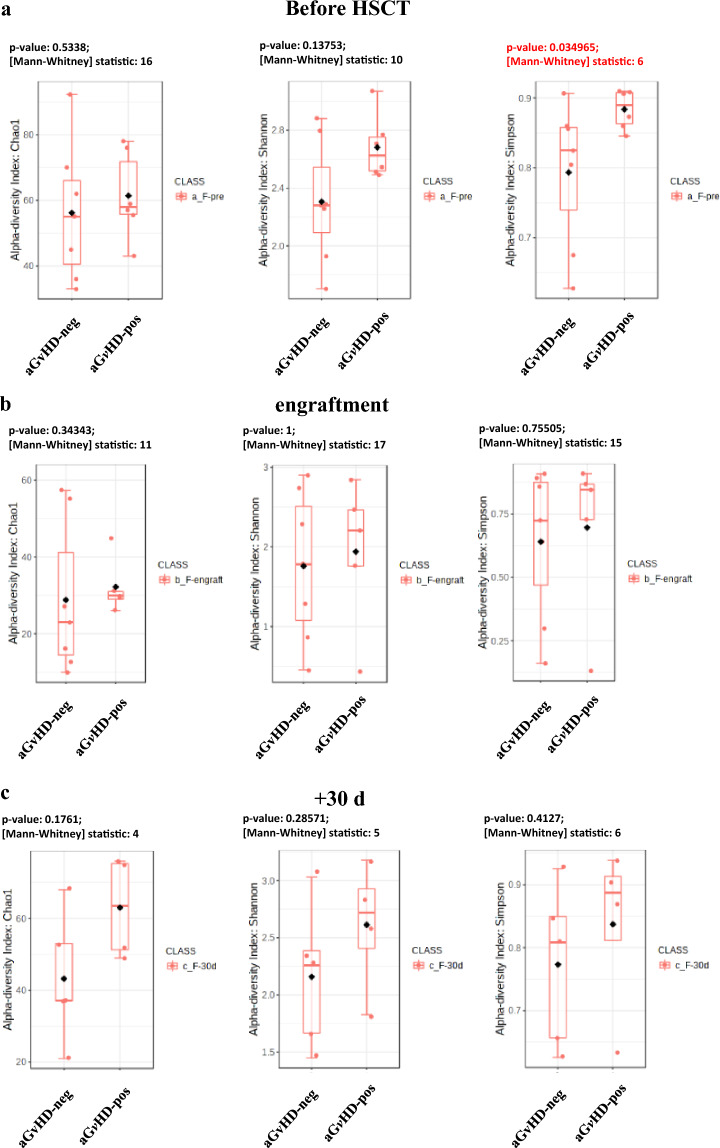
The results show a comparison of the fecal microbiome in patients over 2 years old. The comparison is between the microbiome of samples that underwent cutaneous aG*v*HD and those that never developed such complications. The indexes in the graph estimate community richness (Chao1-index) or community richness and evenness (Shannon or Simpson indexes) from left to right. (**a**) Data before HSCT; (**b**) samples at engraftment; and (**c**) samples at 30 days after transplant.

**Figure 7:**
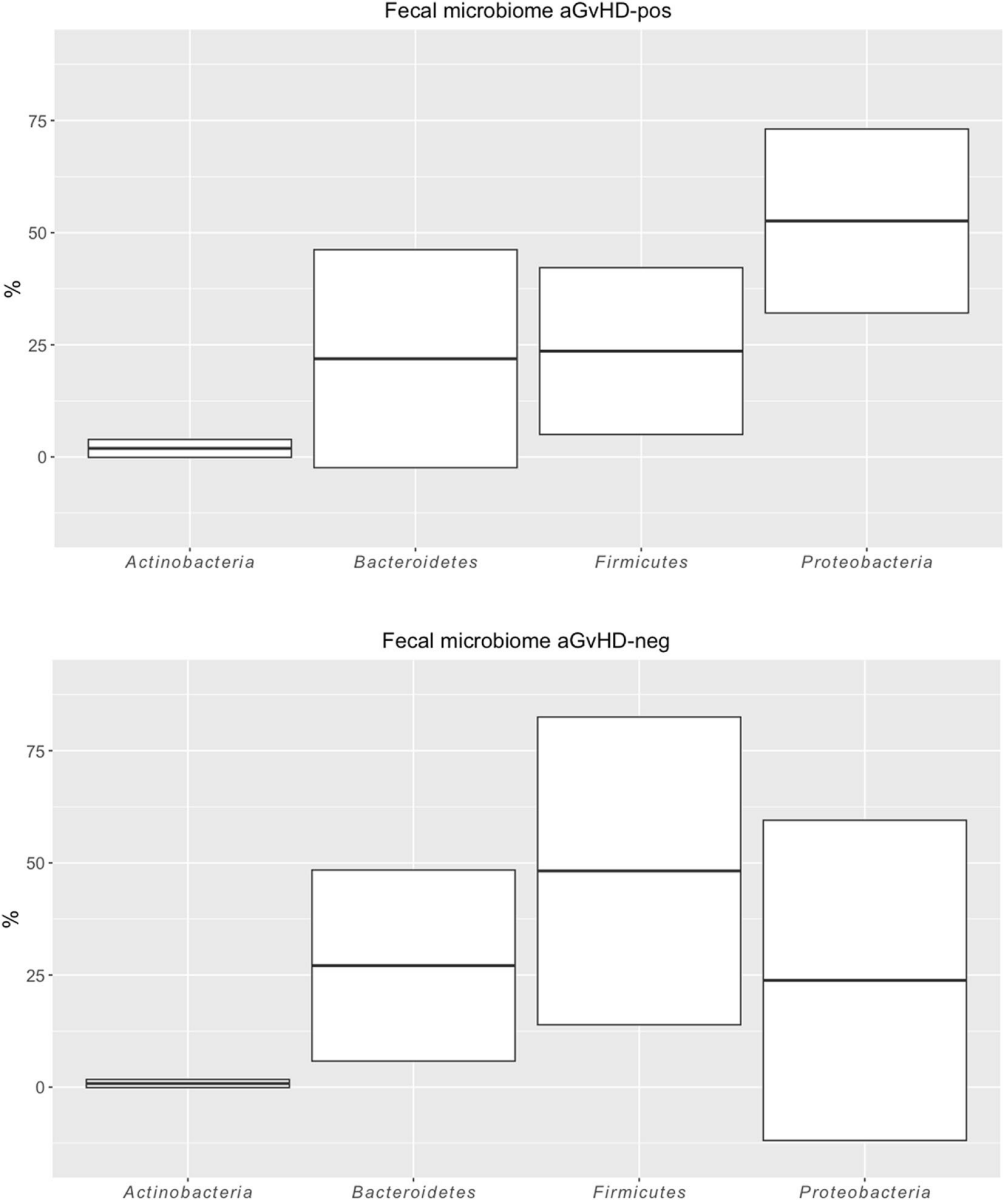
Phila abundances in fecal microbiome samples at engraftment, comparing patients that suffered from acute Graft Versus Host Disease (aG*v*HD) shown on the right with others that never showed such complications (on the left). In square brackets, it was indicated the number of patients belonging to the groups compared in the analysis. A horizontal line splitting each box in two indicates the median. The box limit indicates the upper and lower quartiles of the data.

**Figure 8:**
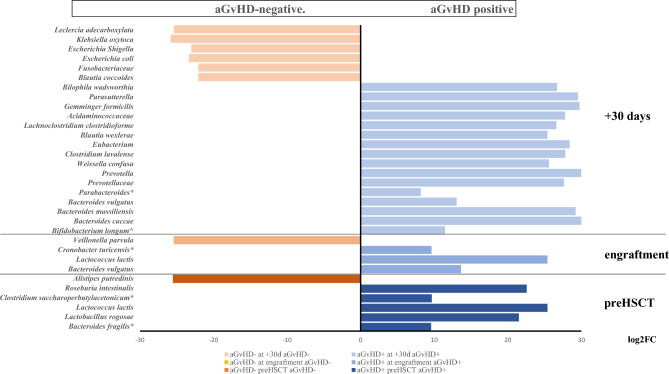
The figure showed the DESeq2 analysis to identify the difference in abundance of various taxa between oral samples that later developed acute GvHD (aGvHD), either before HSCT (6 patients) at engraftment (5 patients) or after 30 days from it (4 patients), and specimens that never had mucositis (7, 7 and 5 patients, respectively). The fold change value, represented as logarithmic on base 2 (log2FC), shows the increase or decrease of abundance of a particular taxa in the comparison between the two groups of samples. FDR (False Discovery Rate) indicates the statistical significance value after adjustment for multiple comparisons. All statistical analyses showed FDR < 0.001, except for the taxa indicated with ^, FDR < 0.01, or with an asterisk (*) that had FDR < 0.05.

**Figure 9:**
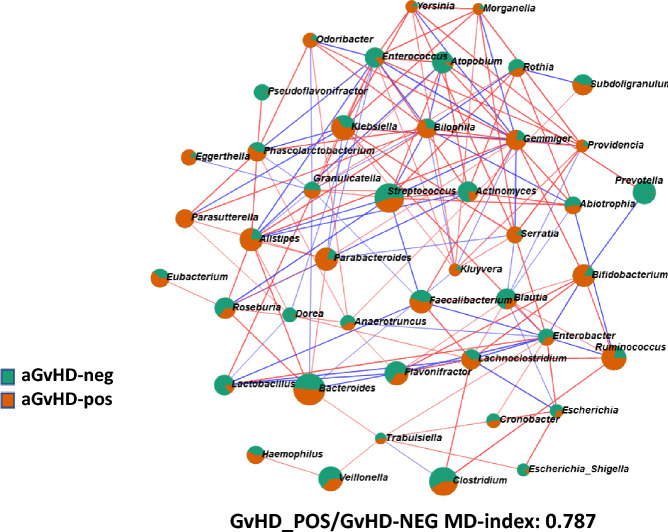
Fecal microbiome from patients treated with myeloablative regimen at HSCT engraftment. Acute Graft Versus Host Disease (aGvHD) positive samples were compared to negative ones for such complications. Each node represents genera that were differently colored in consideration of the preferential abundance in aGvHD samples (orange) or aGvHD-negative samples (green). Red lines connecting nodes indicate a positive correlation between taxa, while blue lines showed a negative one. MD-index represents the dysbiosis-index of positive cases over negative ones. Network was computed for *p* <  = 0.05 and correlation threshold was set at 0.3.

Regarding aG*v*HD, the longitudinal analysis of Chao1, Shannon, and Simpson’s alpha diversity indexes showed higher median values on aG*v*HD samples (Fig. 6). Simpson index was the only measure to reach statistical significance, at least in the pre-HSCT specimens (*p* = 0.0350) (Fig. 6). At engraftment, the compositional microbiome analysis showed a sturdy increase in the relative abundance of *Proteobacteria* in patients with aG*v*HD from 17 to 49% (Fig. 7). More in detail, the *Firmicutes/Proteobacteria* ratio was 3.2 in samples from patients without cutaneous aG*v*HD and 0.5 in cutaneous aG*v*HD patients, thus indicating an unbalanced increase of *Proteobacteria* compared to the *Firmicutes* in aG*v*HD patients. Differently from the patients who developed mucositis, the fecal microbiome of subjects with cutaneous aG*v*HD was associated with the *Proteobacteria* phylum (Fig. 8, Table S6). This data might indicate either a direct dependency of the showed microbial family with aG*v*HD or an association with pro-inflammatory taxa, as suggested by others^22^. More in detail, different genera and species showed a positive correlation with cutaneous aG*v*HD in stool samples, like some species of *Enterobacteriaceae* like *Klebsiella*, *Kluyvera, Yersinia, Serratia,* and *Enterobacter*, as it was also confirmed by the network correlation analysis performed with SPARCC algorithms (Fig. 9). At 30 days after HSCT, among the bacterial taxonomy with higher abundance in stool samples from patients suffering from cutaneous aG*v*HD, different of them belong to the *Alphaproteobacteria (Gemminger formicilis)* and *Deltaproteobacteria* (*Bilophila wadsworthia*) (Fig. 8 and Supplementary material Table S6)*,* followed by the ones belonging to the *Betaproteobacteria* (*Parasutterella*), and *Bacteroides* genus (Fig. 8 and Supplementary material Table S6). Indeed, the *Firmicutes/Bacteroidetes* ratio always showed a lower value in patients with cutaneous aG*v*HD compared to the ones that did not develop it at all the time points studied (Fig. 7).

## Discussion

This study aims to investigate the changes in the microbiome of the oral and intestinal microbiomes in pediatric patients who underwent allogeneic HSCT. The study found that the toxic effects of different conditioning regimens used during HSCT can lead to microbial dysbiosis in various environments, including the oral cavity^23^. This can result in the development of infectious and immunological complications in patients. The study highlights the importance of monitoring the microbiome in HSCT patients, especially in the oral cavity, to prevent complications and ensure successful outcomes. The peculiarity of this study is the detailed analysis of the oral microbiome after HSCT, which is rarely reported in the literature^24^. Compositional analysis of the oral bacterial phyla demonstrated a heavy expansion of *Fusobacteria* in patients with OM. Indeed, samples from patients affected by severe mucositis (grade ≥ 3) showed an abundance of the genus Fusobacterium of 18%, while the same taxa displayed only 2% in patients that never developed OM. All three alpha diversity profiling indexes (Chao1, Shannon, and Simpson) suggested the susceptibility of both oral and fecal microbiomes to the chemo and/or radiotherapy administered during the CR. Indeed, the used indexes had the lowest value at engraftment and reached the highest score 100 days after transplant, as a flourish of microbiota over time. Our data show that a decrease in taxa richness and evenness was associated with CR for oral microbiome analysis, whereas the fecal specimens showed a statistically relevant decrease in richness (Chao-1) only. Longitudinal analysis of the oral and fecal microbiomes showed a mutually exclusive presence of taxa in one of the two studied environments, with few exceptions. In patients affected by oral mucositis, the oral microbiome showed an increased abundance of different species of the *Fusobacterium*, *Mycoplasma*, *Capnocytophaga*, and *Prevotella* and a depletion of *Streptococcus*, *Granulicatella*, and *Rothia* taxa. This is in line with the findings of previous reports^2,20^. In contrast, the gut microbiome showed enrichment of *Eggerthella lenta* and depletion of *Streptococcus*, *Rothia*, Actinomyces, *Granulicatella,* and *Veillonella* species in subjects presenting mucositis (grade ≥ 2). Our data mainly agree with previous reports^2,25^. Moreover, our data on differential abundance and the SparCC network analysis showed an association between the *Atopobium* genus and its species *Atopobium parvulum* with mucositis of grade ≤ 2.

It has been observed that the Atopobium parvulum species has been reported by others to be positively associated with mucositis incidence^26^. It is interesting to note that our data on oral microbiomes indicates that deciphering microbial interactions through network analysis could help identify key bacteria for both health and disease. However, it’s worth mentioning that our findings contradict, at least in part, those of other authors^26^, who have also suggested that the genus *Kingella* is associated with mucositis. In our study, *Kingella* was mainly found in samples from patients who had not experienced oral mucositis. This suggests that further research is needed to fully understand the role of *Kingella* in oral health and disease.

Our data showed that *Alphaproteobacteria (Gemminger),* and *Deltaproteobacteria* (*Bilophila*) were positively associated with aGvHD. The role of *Escherichia*, a member of *Gammaproteobacteria*, in supporting cutaneous aG*v*HD is quite contradictory since our data mainly found a negative correlation with cutaneous aG*v*HD, differently from what has been already reported by other groups^27,28^. Our data could be explained by the effect of some metabolites secreted by *Escherichia coli*, like indole and its derivatives, that were known to mitigate G*v*HD^29^. Also, *Prevotella* descendants and *Bacteroides* were negatively associated with aG*v*HD (Supplementary material Table S6), as suggested by a previous report^22^. However, in an animal model, others found a positive correlation between *Prevotella ssp*. and aG*v*HD^28^. In addition, the behavior of the *Clostridiales* was the opposite of what was already presented^10^, except for the *Anaerostipes* genus, which in our data showed a negative correlation with cutaneous aG*v*HD.

Our research, as well as studies conducted by others, indicate that the imbalance of microbes caused by CR (conditioning regimen) can result in complications during hematopoietic stem cell transplantation (HSCT), such as mucositis and cutaneous acute graft-versus-host disease (aGvHD). To prevent these conditions, several methods have been tested, including the use of probiotics, pre/postbiotics, nutritionally active substances, and various species of lactic acid bacteria^30–33^. These approaches have shown promise in reducing the incidence of CR-induced oral mucositis^33^. Additionally, other studies have identified bacterial taxa associated with the development of mucositis and its severity and the role of dysbiosis during preconditioning^4,34^. It may be possible that studies in this field are paving the way toward more personalized and effective treatments by evaluating bacterial signature profiles might lead to avoiding or decreasing the severity of HSCT complications.

### Supplementary Information

Supplementary Tables.

## Data Availability

The datasets presented in this study are openly available in the Short Read Archive (SRA), Sequence Read Archive NCBI-NIH. SRA_BioProject ID PRJNA929702.

## Abbreviations

HSCT: Hematopoietic stem cell transplantation
OM: Oral mucositis
aG*v*HD: Acute graft versus host disease
G*v*HD: Graft versus host disease
CR: Conditioning regimen
GI: Gastrointestinal
TBI: Total body irradiation
Treo: Treosulfan
FDR: False discovery rate
SparCC: Sparse correlations for compositional data
MD-index: Microbial dysbiosis-index
MAC: Myeloablative regimen
RIC: Reduced intensity conditioning
OTUs: Operational taxonomic units
F/B: *Firmicutes/Bacteroidetes*
F/P: *Firmicutes/Proteobacteri*A
SRA: Short read archive
PCoA: Principal coordinates analysis
