# Supplementary material for: Association between oral and fecal microbiome dysbiosis and treatment complications in pediatric patients undergoing allogeneic hematopoietic stem cell transplantation
Source: Sci Rep. 2024 Mar 20;14:6708. doi: 10.1038/s41598-024-55690-6 (PMC10954761; doi:10.1038/s41598-024-55690-6)
Supplement: Supplementary file 1 — Supplementary Tables. [file 41598_2024_55690_MOESM1_ESM.pdf]

Table S1. Relative abundances of microbial taxa in oral or fecal samples comparing specimens at engraftment with the ones before HSCT.

	ORAL SWABS Engraftment [15] *vs* pre-HSCT [14]					STOOLS Engraftment [13] *vs* pre-HSCT [15]				
**Taxonomy**	**zero-inflated Gaussian fit**	**EdgeR**		**DESeq2**		**zero-inflated Gaussian fit**	**EdgeR**		**DESeq2**	
	FDR	log2FC	FDR	log2FC	FDR	FDR	log2FC	FDR	log2FC	FDR
**Higher abundance at engraftment**										
p_Actinobacteria; c_Actinobacteria; **o_Coriobacteriales**							7.3814	3.4E-5		
p_Actinobacteria; c_Actinobacteria; o_Coriobacteriales; f_Coriobacteriaceae; **g_Atopobium**							8.1258	3.3E-4		
p_Actinobacteria; c_Actinobacteria; o_Coriobacteriales; f_Coriobacteriaceae; **g_Atopobium; s_parvulum**							7.1565	0.0018		
p_Actinobacteria; c_Actinobacteria; **o_Actinomycetales**							7.5201	7.1E-5		
p_Actinobacteria; c_Actinobacteria; o_Actinomycetales; **f_Actinomycetaceae**							6.2149	4.1E-4		
p_Actinobacteria; c_Actinobacteria; o_Actinomycetales; f_Actinomycetaceae; **g_Actinomyces**							6.115	0.0013		
p_Actinobacteria; c_Actinobacteria; o_Actinomycetales; f_Actinomycetaceae; **g_Actinomyces; s_lingnae**		4.2131	0.0022							
p_Actinobacteria; c_Actinobacteria; o_Actinomycetales; f_Actinomycetaceae; **g_Actinomyces; s_odontolyticus**							4.9568	0.0083		
p_Actinobacteria; c_Actinobacteria; o_Actinomycetales; f_Actinomycetaceae; **g_Actinomyces; s_sp.**							5.9498	0.0018		
p_Actinobacteria; c_Actinobacteria; o_Actinomycetales; **f_Corynebacteriaceae**		6.7723	7.3E-4				6.2595	6.7E-5		
p_Actinobacteria; c_Actinobacteria; o_Actinomycetales; f_Corynebacteriaceae; **g_Corynebacterium**		6.0025	0.0055							
p_Actinobacteria; c_Actinobacteria; o_Actinomycetales; f_Corynebacteriaceae; **g_Corynebacterium; s_durum**		5.6943	0.0035							
p_Actinobacteria; c_Actinobacteria; o_Actinomycetales; **f_Micrococcaceae**						0.0089	7.4182	2.5E-5		
p_Actinobacteria; c_Actinobacteria; o_Actinomycetales; f_Micrococcaceae; **g_Rothia**						0.0212	6.5801	4.0E-4		
p_Actinobacteria; c_Actinobacteria; o_Actinomycetales; f_Micrococcaceae; **g_Rothia; s_mucilaginosa**						0.0174	5.613	0.0021		
p_Actinobacteria; **c_Coriobacteria**							6.8245	3.4E-5		
p_Bacteroidetes; c_Bacteroidia; o_Bacteroidales; f_Bacteroidaceae; **g_Bacteroides**		5.5597	0.0127							
p_Bacteroidetes; c_Bacteroidia; o_Bacteroidales; **f_Paraprevotellaceae**		7.526	8.0E-5							
p_Bacteroidetes; c_Bacteroidia; o_Bacteroidales; f_Porphyromonadaceae; **g_Porphyromonas**		2.7608	0.0319							
p_Bacteroidetes; c_Bacteroidia; o_Bacteroidales; f_Porphyromonadaceae; **g_Porphyromonas; s_catoniae**		4.6391	0.0090							
p_Bacteroidetes; c_Bacteroidia; o_Bacteroidales; f_Prevotellaceae; **g_Prevotella; s_copri**		3.2691	0.0377							
p_Bacteroidetes; c_Bacteroidia; o_Bacteroidales; f_Prevotellaceae; **g_Prevotella; s_oulorum**		4.2475	0.020							
p_Bacteroidetes; c_Bacteroidia; o_Bacteroidales; f_Prevotellaceae; **g_Prevotella; s_salivae**		3.774	0.0090							
p_Bacteroidetes; c_Bacteroidia; o_Bacteroidales; **f_Rikenellaceae**							3.7102	0.0249		
**p_Firmicutes**		3.0531	0.0021							
p_Firmicutes; **c_Bacilli**							5.4421	1.2E-5	2.6872	0.0020
p_Firmicutes; c_Bacilli; **o_Bacillales**							4.7501	1.2E-4		
p_Firmicutes; c_Bacilli; o_Bacillales; **f_Bacillales Incertae Sedis**							5.7058	3.7E-5		
p_Firmicutes; c_Bacilli; o_Bacillales; f_Bacillales Incertae Sedis; **g_Gemella**							3.9403	0.0065		
p_Firmicutes; c_Bacilli; o_Bacillales; f_Bacillales Incertae Sedis; **g_Gemella; s_haemolysans**							3.814	0.0037		
p_Firmicutes; c_Bacilli; o_Bacillales; f_Bacillales Incertae Sedis; **g_Gemella; s_morbillorum**		4.9011	0.0090							
p_Firmicutes; c_Bacilli; **o_Lactobacillales**		2.4712	0.0478				4.9931	9.0E-5	2.3199	0.0257
p_Firmicutes; c_Bacilli; o_Lactobacillales; **f_Aerococcaceae**		4.9263	0.0156				9.2371	2.5E-5		
p_Firmicutes; c_Bacilli; o_Lactobacillales; f_Aerococcaceae; **g_Abiotrophia**		4.3085	0.0142				9.7647	4.1E-5		
p_Firmicutes; c_Bacilli; o_Lactobacillales; f_Aerococcaceae; **g_Abiotrophia; s_defectiva**							8.0451	5.5E-4		
p_Firmicutes; c_Bacilli; o_Lactobacillales; **f_Carnobacteriaceae**							4.2753	0.0070		
p_Firmicutes; c_Bacilli; o_Lactobacillales; f_Carnobacteriaceae; **g_Granulicatella**							3.8507	0.0118		
p_Firmicutes; c_Bacilli; o_Lactobacillales; f_Carnobacteriaceae; **g_Granulicatella; s_elegans**							3.3586	0.0229		
p_Firmicutes; c_Bacilli; o_Lactobacillales; **f_Streptococcaceae**							6.4836	2.5E-5		
p_Firmicutes; c_Bacilli; o_Lactobacillales; f_Streptococcaceae; **g_Streptococcus**		2.299	0.0371				4.9109	4.8E-4		
p_Firmicutes; c_Bacilli; o_Lactobacillales; f_Streptococcaceae; **g_Streptococcus; s_infantis**							5.0294	0.0018		
p_Firmicutes; c_Bacilli; o_Lactobacillales; f_Streptococcaceae; **g_Streptococcus; s_mitis**		3.0214	0.0170			0.0049	6.8733	2.3E-4		
p_Firmicutes; c_Bacilli; o_Lactobacillales; f_Streptococcaceae; **g_Streptococcus; s_pneumoniae**						0.0046	5.5241	2.2E-4		
p_Firmicutes; **c_Clostridia**		2.5477	0.0171							
p_Firmicutes; c_Clostridia; **o_Clostridiales**		1.9438	0.0478							
p_Firmicutes; c_Clostridia; o_Clostridiales; f_Lachnospiraceae; **g_Catonella**	0.0176	3.2883	0.0142							
p_Firmicutes; c_Clostridia; o_Clostridiales; **f_Clostridiales Family XIII_Incertae Sedis**							5.9783	3.4E-4		
p_Firmicutes; c_Clostridia; o_Clostridiales; **f_Peptostreptococcaceae**		3.4472	0.0433				3.9408	0.0070		
p_Firmicutes; c_Clostridia; o_Clostridiales; f_Peptostreptococcaceae; **g_Peptostreptococcus**		3.2159	0.0451							

p_Firmicutes; c_Clostridia; o_Clostridiales; f_Peptostreptococcaceae; **g_Peptostreptococcus; s_stomatis**		3.2146	0.0471							
p_Firmicutes; c_Clostridia; o_Clostridiales; **f_Ruminococcaceae**		6.0552	4.4E-4							
p_Firmicutes; **c_Negativicutes**		3.1565	0.0171							
p_Firmicutes; c_Negativicutes; **o_Selenomonadales**		4.9278	0.0012							
p_Firmicutes; c_Negativicutes; o_Selenomonadales; **f_Veillonellaceaea**		4.398	0.0017							
p_Firmicutes; c_Negativicutes; o_Selenomonadales; f_Veillonellaceaea; **g_Veillonella**		2.885	0.0253							
p_Firmicutes; c_Negativicutes; o_Selenomonadales; f_Veillonellaceaea; **g_Veillonella; s_alcalescens**		5.3071	0.0016							
p_Firmicutes; c_Negativicutes; o_Selenomonadales; f_Veillonellaceaea; **g_Veillonella; s_parvula**		4.2496	0.0090							
p_Firmicutes; c_Negativicutes; o_Selenomonadales; f_Veillonellaceaea; **g_Veillonella; s_sp.**		3.249	0.0249							
p_Fusobacteria; c_Fusobacteriia; o_Fusobacteriales; f_Fusobacteriaceae; **g_Fusobacterium; s_nucleatum**							5.2901	0.0165		
p_Fusobacteria; c_Fusobacteriia; o_Fusobacteriales; f_Leptotrichiaceae; **g_Leptotrichia; s_buccalis**		3.5536	0.0249							
**p_Proteobacteria**										
p_Proteobacteria; c_Betaproteobacteria; o_Neisseriales; f_Neisseriaceae; **g_Eikenella; s_corrodens**		6.9365	0.0014							
p_Proteobacteria; c_Epsilonproteobacteria; **o_Campylobacterales**		2.5412	0.0438							
p_Proteobacteria; c_Epsilonproteobacteria; o_Campylobacterales; f_Campylobacteraceae; **g_Campylobacter**		3.3341	0.0142							
p_Proteobacteria; c_Epsilonproteobacteria; o_Campylobacterales; f_Campylobacteraceae; **g_Campylobacter; s_gracilis**		7.6857	6.6E-4							
p_Proteobacteria; c_Gammaproteobacteria; o_Enterobacteriales; **f_Enterobacteriaceae**		3.612	0.0181							
p_Proteobacteria; c_Gammaproteobacteria; o_Enterobacteriales; f_Enterobacteriaceae; **g_Serratia**		4.8047	0.0127							
p_Proteobacteria; c_Gammaproteobacteria; o_Enterobacteriales; f_Enterobacteriaceae; **g_Escherichia**							2.8716	0.0452		
**Higher abundance in pre-HSCT**										
p_Actinobacteria; c_Actinobacteria; o_Actinomycetales; f_Actinomycetaceae; **g_Actinomyces; s_viscosus**		4.5037	0.0076							
p_Actinobacteria; c_Actinobacteria; **o_Bifidobacteriales**							5.1686	0.0013		
p_Actinobacteria; c_Actinobacteria; o_Bifidobacteriales; **f_Bifidobacteriaceae**							3.7774	0.0249		
p_Actinobacteria; c_Actinobacteria; o_Bifidobacteriales; f_Bifidobacteriaceae; **g_Bifidobacterium**						0.0098	4.9452	0.0030		
p_Actinobacteria; c_Actinobacteria; o_Bifidobacteriales; f_Bifidobacteriaceae; **g_Bifidobacterium; s_adolescentis**							3.6103	0.0148		
p_Actinobacteria; c_Actinobacteria; o_Bifidobacteriales; f_Bifidobacteriaceae; **g_Bifidobacterium; s_longum**						0.0068	5.9453	0.0021	6.6953	0.0296
p_Actinobacteria; c_Actinobacteria; o_Coriobacteriales; f_Coriobacteriaceae; **g_Atopobium**	0.0043									
p_Bacteroidetes; c_Bacteroidia; o_Bacteroidales; f_Bacteroidaceae; **g_Bacteroides; s_caccae**						0.0068				
p_Bacteroidetes; c_Bacteroidia; o_Bacteroidales; f_Bacteroidaceae; **g_Bacteroides; s_sp.**						0.0049	2.7689	0.0106	9.052	0.0103
p_Bacteroidetes; c_Bacteroidia; o_Bacteroidales; f_Porphyromonadaceae; **g_Tannerella**	0.0028	3.2731	0.0142							
p_Bacteroidetes; c_Bacteroidia; o_Bacteroidales; f_Porphyromonadaceae; **g_Tannerella; s_sp.**	0.0095	3.9979	0.0022	6.9824	0.0248					
p_Bacteroidetes; c_Flavobacteriia; o_Flavobacteriales; f_Flavobacteriaceae; **g_Capnocytophaga; s_gingivalis**	0.0103									
p_Bacteroidetes; c_Bacteroidia; o_Bacteroidales; f_Porphyromonadaceae; **g_Odoribacter; s_splanchnicus**						0.0053				
p_Bacteroidetes; c_Bacteroidia; o_Bacteroidales; **f_Prevotellaceae**						0.0161	6.009	0.0020	6.5734	0.0125
p_Bacteroidetes; c_Bacteroidia; o_Bacteroidales; f_Prevotellaceae; **g_Prevotella**							8.8033	3.3E-4		
p_Bacteroidetes; c_Bacteroidia; o_Bacteroidales; f_Prevotellaceae; **g_Prevotella; s_loescheii**		3.8352	0.0052							
p_Bacteroidetes; c_Bacteroidia; o_Bacteroidales; f_Prevotellaceae; **g_Prevotella; s_sp.**	0.0095	2.9558	0.0093							
p_Firmicutes; c_Bacilli; o_Lactobacillales; f_Streptococcaceae; **g_Streptococcus; s_australis**		3.2128	0.0249							
p_Firmicutes; c_Bacilli; o_Lactobacillales; f_Streptococcaceae; **g_Streptococcus; s_caballi**	0.0024	3.2416	0.0430							
p_Firmicutes; c_Bacilli; o_Lactobacillales; f_Streptococcaceae; **g_Streptococcus; s_lactarius**	0.0383									
p_Firmicutes; c_Bacilli; o_Lactobacillales; f_Streptococcaceae; **g_Streptococcus; s_pseudopneomoniae**							2.8124	0.0407		
p_Firmicutes; c_Bacilli; o_Lactobacillales; f_Streptococcaceae; **g_Streptococcus; s_salivarius**		3.5608	0.0202							
p_Firmicutes; c_Bacilli; o_Lactobacillales; f_Streptococcaceae; **g_Streptococcus; s_sanguinis**	0.0340	5.3729	0.0016	5.7741	0.0110					
p_Firmicutes; c_Bacilli; o_Lactobacillales; f_Streptococcaceae; **g_Streptococcus; s_sinensis**		3.4473	0.0168							
p_Firmicutes; c_Clostridia; o_Clostridiales; f_Clostridiaceae; **g_Clostridium; s_clostridiofrome**							4.3756	0.0106		
p_Firmicutes; c_Clostridia; o_Clostridiales; f_Lachnospiraceae; **g_Blautia**						0.0212	4.7941	0.0031		
p_Firmicutes; c_Clostridia; o_Clostridiales; f_Lachnospiraceae; **g_Blautia; s_producta**							4.5759	0.0083		
p_Firmicutes; c_Clostridia; o_Clostridiales; f_Lachnospiraceae; **g_Dorea**						0.0118	5.5878	4.0E-4		
p_Firmicutes; c_Clostridia; o_Clostridiales; f_Lachnospiraceae; **g_Lachnoclostridium; s_clostridiofrome**							4.9674	0.0049		
p_Firmicutes; c_Clostridia; o_Clostridiales; f_Lachnospiraceae; **g_Oribacterium**	0.0066									
p_Firmicutes; c_Clostridia; o_Clostridiales; f_Lachnospiraceae; **g_Oribacterium; s_sinus**	0.0029	5.3088	2.0E-5							
p_Firmicutes; c_Clostridia; o_Clostridiales; f_Lachnospiraceae; **g_Roseburia**						0.0247	3.142	0.0412		
p_Firmicutes; c_Clostridia; o_Clostridiales; f_Lachnospiraceae; **g_Roseburia; s_faecis**						0.0247				
p_Firmicutes; c_Clostridia; o_Clostridiales; f_Lachnospiraceae; **g_Roseburia; s_inulinivorans**						0.0017	5.9441	1.4E-4	8.5624	0.0270
p_Firmicutes; c_Clostridia; o_Clostridiales; f_Lachnospiraceae; **g_[Ruminococcus]; s_torques**						1.3E-4	4.0804	0.0044		
p_Firmicutes; c_Clostridia; o_Clostridiales; f_Lachnospiraceae; **g_Stomatobaculum**	0.0043	2.7883	0.0142							
p_Firmicutes; c_Clostridia; o_Clostridiales; f_Lachnospiraceae; **g_Stomatobaculum; s_longum**		2.7234	0.0090							
p_Firmicutes; c_Clostridia; o_Clostridiales; f_Ruminococcaceae; **g_Faecalibacterium**							4.4003	0.0031		
p_Firmicutes; c_Clostridia; o_Clostridiales; f_Ruminococcaceae; **g_Faecalibacterium; s_prausnitzii**						0.0068	3.6383	0.0276		

p_Firmicutes; c_Clostridia; o_Clostridiales; f_Eubacteriaceae; ***g_Eubacterium***						0.0354	3.5669	0.0118		
p_Firmicutes; c_Clostridia; o_Clostridiales; f_Eubacteriaceae; ***g_Eubacterium; s_eligens***							4.504	0.0037		
p_Firmicutes; c_Clostridia; o_Clostridiales; f_Unclassified Clostridiales; ***g_Pseudoflavonifractor***							5.0745	0.0048		
p_Firmicutes; c_Erysipelotrichia; o_Erysipelotrichales; f_Erysipelotrichaceae; ***g_Bulleidia***	0.0028									
p_Firmicutes; c_Negativicutes; o_Selenomonadales; f_Veillonellaceaea; ***g_Megasphaera***	0.0312									
p_Proteobacteria; c_Alphaproteobacteria; ***o_Rhizobiales***						0.0067				
p_Proteobacteria; c_Alphaproteobacteria; o_Rhizobiales; ***f_Hyphomicrobiaceae***							2.866	0.0490		
p_Proteobacteria; c_Alphaproteobacteria; o_Rhizobiales; f_Hyphomicrobiaceae; ***g_Gemminger***						0.0212				
p_Proteobacteria; c_Alphaproteobacteria; o_Rhizobiales; f_Hyphomicrobiaceae; ***g_Gemminger; s_formicilis***						0.0120				
p_Proteobacteria; c_Betaproteobacteria; o_Neisseriales; f_Neisseriaceae; ***g_Neisseria; s_perflava***	0.0024									
p_Proteobacteria; c_Gammaproteobacteria; o_Pasteurellales; f_Pasteurellaceae; ***g_Mannheimia; s_varigena***						8.8E-4	5.889	1.8E-4	24.908	1.1E-17

This table showed the comparison of differential abundance of oral (in the middle columns) or fecal (on the right columns) samples between engraftment and pre-HSCT time points. In square brackets, it was indicated the number of patients belonging to the groups compared in the analysis. The differential abundance statistical analysis for microbial taxa used different algorythms (zero-inflated Gaussian fit, EdgeR, or DESeq2). The complete taxonomy is indicated on the left column (*p_Phylum; c_Class; o_Order; f_Family; g_Genus; s_Species*). **FDR** (False Discovery Rate) indicates the statistical significance value after adjustment for multiple comparisons. The fold change value indicated as logarithmic on base 2 (log2FC) represents how much is the increase/decrease of abundance of a particular taxa in the comparisons between the two group of samples. The FDR shown had values equal to or less than 0.05 and were considered statistically significant.

Table S2. Relative abundances of microbial taxa in oral or fecal samples comparing specimens after 30 days from transplant with the ones before HSCT.

	ORAL SWABS +30d [10] *vs* pre-HSCT [14]					STOOLS +30d [10] *vs* pre-HSCT [15]				
**Taxonomy**	**zero-inflated Gaussian fit**	**EdgeR**		**DESeq2**		**zero-inflated Gaussian fit**	**EdgeR**		**DESeq2**	
	FDR	log2FC	FDR	log2FC	FDR	FDR	log2FC	FDR	log2FC	FDR
**Higher abundance at +30d**										
p_Actinobacteria; c_Actinobacteria; o_Actinomycetales; f_Actinomycetaceae; ***g_Actinomyces***							4.2357	0.0173		
p_Actinobacteria; c_Actinobacteria; o_Actinomycetales; f_Actinomycetaceae; ***g_Actinomyces; s_lingnae***		3.9866	0.0047							
p_Actinobacteria; c_Actinobacteria; o_Actinomycetales; f_Actinomycetaceae; ***g_Actinomyces; s_sp.***							4.7395	0.0060		
p_Actinobacteria; c_Actinobacteria; o_Actinomycetales; ***f_Micrococcaceae***							8.2557	0.0055		
p_Actinobacteria; c_Actinobacteria; o_Actinomycetales; f_Micrococcaceae; ***g_Rothia***							6.9637	4.8E-4		
p_Actinobacteria; c_Actinobacteria; o_Actinomycetales; f_Micrococcaceae; ***g_Rothia; s_mucillaginosa***							5.7294	0.0047		
p_Bacteroidetes; c_Bacteroidia; o_Bacteroidales; f_Bacteroidaceae; ***g_Bacteroides; s_caccae***							5.8149	0.0055		
p_Bacteroidetes; c_Bacteroidia; o_Bacteroidales; f_Bacteroidaceae; ***g_Bacteroides; s_ovatus***							6.1213	0.0055		
p_Bacteroidetes; c_Bacteroidia; o_Bacteroidales; f_Porphyromonadaceae; ***g_Porphyromonas; s_catoniae***		3.8285	0.0274							
p_Bacteroidetes; c_Bacteroidia; o_Bacteroidales; ***f_Paraprevotellaceae***		3.1148	0.0472							
p_Bacteroidetes; c_Bacteroidia; o_Bacteroidales; f_Prevotellaceae; ***g_Prevotella; s_oris***		4.3226	0.0141							
p_Firmicutes		6.9972	4E-7							
p_Firmicutes; ***c_Bacilli***		3.1983	0.0209							
p_Firmicutes; c_Bacilli; ***o_Bacillales***							10.07	6.3E-5		
p_Firmicutes; c_Bacilli; o_Bacillales; ***f_Bacillales Incertae Sedis***		2.8471	0.0472				11.292	1.1E-4		
p_Firmicutes; c_Bacilli; o_Bacillales; f_Bacillales Incertae Sedis; ***g_Gemella***		3.8737	0.0052				8.2428	3.1E-5		
p_Firmicutes; c_Bacilli; o_Bacillales; f_Bacillales Incertae Sedis; ***g_Gemella; s_haemolysans***							8.0751	1.0E-4		
p_Firmicutes; c_Bacilli; o_Bacillales; f_Bacillales Incertae Sedis; ***g_Gemella; s_morbillorum***		7.1907	9.9E-4							
p_Firmicutes; c_Bacilli; o_Lactobacillales; ***f_Aerococcaceae***							7.3303	0.0123		
p_Firmicutes; c_Bacilli; o_Lactobacillales; f_Aerococcaceae; ***g_Abiotrophia***							8.8636	7.5E-5		
p_Firmicutes; c_Bacilli; o_Lactobacillales; f_Aerococcaceae; ***g_Abiotrophia; s_defectiva***							7.5311	0.0015		
p_Firmicutes; c_Bacilli; o_Lactobacillales; ***f_Carnobacteriaceae***							7.935	0.0055		
p_Firmicutes; c_Bacilli; o_Lactobacillales; f_Carnobacteriaceae; ***g_Granulicatella***							6.1871	9.6E-4		
p_Firmicutes; c_Bacilli; o_Lactobacillales; f_Carnobacteriaceae; ***g_Granulicatella; s_adiacens***							5.2127	0.0055		
p_Firmicutes; c_Bacilli; o_Lactobacillales; f_Carnobacteriaceae; ***g_Granulicatella; s_elegans***							5.7962	0.0024		
p_Firmicutes; c_Bacilli; o_Lactobacillales; ***f_Streptococcaceae***		2.674	0.0472				4.5853	0.0256		
p_Firmicutes; c_Bacilli; o_Lactobacillales; f_Streptococcaceae; ***g_Streptococcus***		2.9748	0.0273				5.0158	9.6E-4		
p_Firmicutes; c_Bacilli; o_Lactobacillales; f_Streptococcaceae; ***g_Streptococcus; s_mitis***							3.9291	0.0103		
p_Firmicutes; c_Bacilli; o_Lactobacillales; f_Streptococcaceae; ***g_Streptococcus; s_oralis***		4.1756	0.0141							
p_Firmicutes; c_Bacilli; o_Lactobacillales; f_Streptococcaceae; ***g_Streptococcus; s_pseudopneomoniae***							4.3441	0.0058		
p_Firmicutes; c_Bacilli; o_Lactobacillales; f_Streptococcaceae; ***g_Streptococcus; s_salivarius***							4.3928	0.0129		
p_Firmicutes; c_Bacilli; o_Lactobacillales; f_Streptococcaceae; ***g_Streptococcus; s_sanguinis***							4.6339	0.0024		
p_Firmicutes; ***c_Clostridia***							4.4046	0.0089		
p_Firmicutes; c_Clostridia; o_Clostridiales; f_Clostridiaceae; ***g_Clostridium; s_asparagiforme***							4.7785	0.0015		
p_Firmicutes; c_Clostridia; o_Clostridiales; f_Clostridiaceae; ***g_Clostridium; s_lavalense***							3.8402	0.0140		
p_Firmicutes; c_Clostridia; o_Clostridiales; ***f_Clostridiales Family XIII_Incertae Sedis***							5.6774	0.0046		
p_Firmicutes; c_Clostridia; o_Clostridiales; f_Lachnospiraceae; ***g_Catonella***		4.178	0.0038							
p_Firmicutes; c_Clostridia; o_Clostridiales; f_Lachnospiraceae; ***g_Catonella; s_morbi***		4.3615	0.0047							
p_Firmicutes; c_Clostridia; o_Clostridiales; ***f_Peptostreptococcaceae***		3.5202	0.0472							
p_Firmicutes; c_Clostridia; o_Clostridiales; f_Lachnospiraceae; ***g_[Ruminococcus]; s_gnavus***							4.5976	0.0054		
p_Firmicutes; c_Clostridia; o_Clostridiales; f_Unclassified Clostridiales; ***g_Pseudoflavonifractor***							6.2274	9.6E-4		
p_Firmicutes; ***c_Erysipelotrichia***							3.6582	0.0089		
p_Firmicutes; c_Erysipelotrichia; o_Erysipelotrichales; ***f_Erysipelotrichaceae***							4.2825	0.0151		
p_Firmicutes; ***c_Negativicutes***		8.1071	1.9E-6							
p_Firmicutes; c_Negativicutes; ***o_Selenomonadales***		7.7693	2.8E-6							
p_Firmicutes; c_Negativicutes; o_Selenomonadales; ***f_Veillonellaceaea***		7.0736	4.4E-5							
p_Firmicutes; c_Negativicutes; o_Selenomonadales; f_Veillonellaceaea; ***g_Veillonella***		6.5141	2.7E-4							
p_Firmicutes; c_Negativicutes; o_Selenomonadales; f_Veillonellaceaea; ***g_Veillonella; s_alcalescens***		8.5441	1.7E-4							

p_Firmicutes; c_Negativicutes; o_Selenomonadales; f_Veillonellaceaea; **g_Veillonella; s_parvula**		6.87	0.0010							
p_Firmicutes; c_Negativicutes; o_Selenomonadales; f_Veillonellaceaea; **g_Veillonella; s_rogosae**		5.0833	0.0207							
p_Proteobacteria; c_Epsilonproteobacteria; o_Campylobacterales; f_Campylobacteraceae; **g_Campylobacter; s_gracilis**	0.0385	3.803	0.0141							
p_Proteobacteria; c_Alphaproteobacteria; **o_Sphingomonadales**							3.6893	0.0253		
p_Proteobacteria; c_Alphaproteobacteria; o_Sphingomonadales; **f_Sphingomonadaceae**							4.5059	0.0055		
p_Proteobacteria; c_Gammaproteobacteria; o_Enterobacteriales; **f_Enterobacteriaceae**		3.8338	0.0420							
p_Proteobacteria; c_Gammaproteobacteria; o_Enterobacteriales; f_Enterobacteriaceae; **g_Serratia**	0.0388	5.4969	0.0041							
p_Proteobacteria; c_Gammaproteobacteria; o_Enterobacteriales; f_Enterobacteriaceae; **g_Klebsiella; s_sp.**						0.0190	4.7641	0.0058		
p_Proteobacteria; c_Gammaproteobacteria; o_Pasteurellales; **f_Pasteurellaceae**							7.0956	0.0123		
p_Proteobacteria; c_Gammaproteobacteria; o_Pasteurellales; f_Pasteurellaceae; **g_Haemophilus**							6.257	0.0023		
p_Proteobacteria; c_Gammaproteobacteria; o_Pasteurellales; f_Pasteurellaceae; **g_Haemophilus; s_parainfluenzae**							5.7307	0.0060		
p_Proteobacteria; c_Gammaproteobacteria; o_Pasteurellales; f_Pasteurellaceae; **g_Mannheimia; s_varigena**							4.1841	0.0221		
**Higher abundance in pre-HSCT**										
p_Actinobacteria; c_Actinobacteria; o_Bifidobacteriales; f_Bifidobacteriaceae; **g_Bifidobacterium; s_longum**							5.4169	0.0218		
p_Actinobacteria; c_Actinobacteria; o_Coriobacteriales; f_Coriobacteriaceae; **g_Atopobium**							3.6067	0.0298		
p_Actinobacteria; c_Actinobacteria; o_Actinomycetales; f_Propionibacteriaceae; **g_Propionibacterium**	0.0388									
p_Actinobacteria; c_Actinobacteria; o_Actinomycetales; f_Micrococcaceae; **g_Rothia; s_aeria**	0.0385	6.4473	0.0107							
p_Actinobacteria; c_Actinobacteria; o_Actinomycetales; f_Micrococcaceae; **g_Rothia; s_dentocariosa**	0.0385	5.4283	0.0141							
p_Bacteroidetes; c_Bacteroidia; o_Bacteroidales; **f_Porphyromonadaceae**							3.8555	0.0287		
p_Bacteroidetes; c_Bacteroidia; o_Bacteroidales; f_Porphyromonadaceae; **g_Parabacteroides**							4.2481	0.0434		
p_Bacteroidetes; c_Bacteroidia; o_Bacteroidales; f_Porphyromonadaceae; **g_Parabacteroides; s_merdae**						0.0045	8.0334	0.0012	24.911	4.7E-21
p_Bacteroidetes; c_Bacteroidia; o_Bacteroidales; f_Bacteroidaceae; **g_Bacteroides; s_finegoldii**						0.0203	7.7279	0.0024		
p_Bacteroidetes; c_Bacteroidia; o_Bacteroidales; f_Bacteroidaceae; **g_Bacteroides; s_fragilis**							4.6922	0.0424	9.2376	0.0480
p_Bacteroidetes; c_Bacteroidia; o_Bacteroidales; f_Bacteroidaceae; **g_Bacteroides; s_vulgatus**							5.4946	0.0115		
p_Bacteroidetes; c_Bacteroidia; o_Bacteroidales; **f_Prevotellaceae**							7.7082	0.0055		
p_Bacteroidetes; c_Bacteroidia; o_Bacteroidales; f_Prevotellaceae; **g_Prevotella**							8.6436	0.0056	9.8118	0.0044
p_Firmicutes; c_Clostridia; o_Clostridiales; f_Eubacteriaceae; **g_Eubacterium**						0.0074	5.8438	0.0048		
p_Firmicutes; c_Clostridia; o_Clostridiales; **f_Oscillospiraceae**							4.0501	0.0126		
p_Firmicutes; c_Clostridia; o_Clostridiales; f_Lachnospiraceae; **g_Dorea**						0.0074	5.8995	0.0048		
p_Firmicutes; c_Clostridia; o_Clostridiales; f_Lachnospiraceae; **g_Lachnoclostridium; s_hathewayi**						0.0190	3.4832	0.0164		
p_Firmicutes; c_Clostridia; o_Clostridiales; f_Lachnospiraceae; **g_Stomatobaculum**	0.0443									
p_Firmicutes; c_Clostridia; o_Clostridiales; f_Lachnospiraceae; **g_Roseburia; s_faecis**						0.0329	4.8301	0.0087		
p_Firmicutes; c_Clostridia; o_Clostridiales; f_Lachnospiraceae; **g_[Ruminococcus]; s_torques**						4.2E-4	5.2939	0.0058		
p_Firmicutes; c_Clostridia; o_Clostridiales; f_Clostridiaceae; **g_Clostridium; s_spiroforme**						0.0249	4.8965	0.0294		
p_Firmicutes; c_Clostridia; o_Clostridiales; f_unclassified Clostridiales; **g_Flavonifractor**						0.0311				
p_Firmicutes; c_Clostridia; o_Clostridiales; f_Ruminococcaceae; **g_Ruminococcus; s_sp.**						0.0190	8.1653	0.0015		
p_Firmicutes; c_Clostridia; o_Clostridiales; f_Ruminococcaceae; **g_Ruminococcus; s_torques**						0.0108	5.0657	0.0097		
p_Proteobacteria; c_Betaproteobacteria; o_Neisseriales; f_Neisseriaceae; **g_Neisseria; s_perflava**		5.673	0.0141							
p_Proteobacteria; c_Betaproteobacteria; o_Burkholderiales; **f_Sutterellaceae**							3.4739	0.0474		
p_Proteobacteria; c_Deltaproteobacteria; o_Desulfovibrionales; **f_Desulfovibrionaceae**							5.3142	0.0110		
p_Proteobacteria; c_Epsilonproteobacteria; o_Campylobacterales; f_Campylobacteraceae; **g_Campylobacter**						0.0311				
p_Proteobacteria; c_Gammaproteobacteria; o_Enterobacteriales; f_Enterobacteriaceae; **g_Cronobacter; s_turicensis**						0.0186				

This table showed the comparison of differential abundance of oral (in the middle columns) or fecal (on the right columns) samples between samples after 30 days from transplant and pre-HSCT time points. In square brackets, it was indicated the number of patients belonging to the groups compared in the analysis. The differential abundance statistical analysis for microbial taxa used different algorythms (zero-inflated Gaussian fit, EdgeR, or DESeq2). The complete taxonomy is indicated on the left column (*p_Phylum; c_Class; o_Order; f_Family; g_Genus; s_Species*). **FDR** (False Discovery Rate) indicates the statistical significance value after adjustment for multiple comparisons. The fold change value indicated as logarithmic on base 2 (log2FC) represents how much is the increase/decrease of abundance of a particular taxa in the comparisons between the two group of samples. The FDR shown had values equal to or less than 0.05 and were considered statistically significant.

Table S3. Relative abundances of microbial taxa in oral or fecal samples comparing specimens after 100 days from transplant with the ones before HSCT.

Taxonomy	ORAL SWABS +100d [5] *vs* pre-HSCT [14]					STOOLS +100d [5] *vs* pre-HSCT [15]				
	zero-inflated Gaussian fit	EdgeR		DESeq2		zero-inflated Gaussian fit	EdgeR		DESeq2	
	FDR	log2FC	FDR	log2FC	FDR	FDR	log2FC	FDR	log2FC	FDR
**Higher abundance at +100d**										
p_Actinobacteria; c_Actinobacteria; o_Actinomycetales; **f_Corynebacteriaceae**		5.5691	0.0014							
p_Actinobacteria; c_Actinobacteria; o_Actinomycetales; f_Corynebacteriaceae; **g_Corinebacterium**		5.4191	0.0017							
p_Actinobacteria; c_Actinobacteria; o_Actinomycetales; f_Corynebacteriaceae; **g_Corinebacterium; s_durum**		4.7808	0.0183							
p_Bacteroidetes; c_Bacteroidia; o_Bacteroidales; f_Prevotellaceae; **g_Prevotella; s_aurantiaca**				21.58	4.5E-10					
p_Firmicutes; c_Clostridia; o_Clostridiales; f_Lachnospiraceae; **g_Lachnoanaerobaculum**		6.1869	1.1E-4							
p_Firmicutes; c_Clostridia; o_Clostridiales; f_Lachnospiraceae; **g_Lachnoanaerobaculum; s_sp.**		5.6685	0.0053							
p_Firmicutes; c_Clostridia; o_Clostridiales; f_Lachnospiraceae; **g_Lachnoanaerobaculum; s_umeaense**		4.174	0.0305							
p_Firmicutes; c_Negativicutes; o_Selenomonadales; f_Veillonellaceaea; **g_Veillonella; s_alcalescens**	0.0195	4.7465	0.0053							
p_Proteobacteria; c_Epsilonproteobacteria; o_Campylobacterales; f_Campylobacteraceae; **g_Campylobacter**		4.3443	0.0039							
p_Proteobacteria; c_Epsilonproteobacteria; o_Campylobacterales; f_Campylobacteraceae; **g_Campylobacter; s_gracilis**	6.3E-4	8.1364	1.8E-5	8.587	9.4E-4					
**Higher abundance in pre-HSCT**										
p_Actinobacteria; c_Actinobacteria; o_Coriobacteriales; f_Coriobacteriaceae; **g_Atopobium**	3.7E-4									
p_Actinobacteria; c_Actinobacteria; o_Actinomycetales; f_Propionibacteriaceae; **g_Propionibacterium**	0.0062									
p_Bacteroidetes; c_Bacteroidia; o_Bacteroidales; f_Porphyromonadaceae; **g_Porphyromonas**	0.0312									
p_Firmicutes; c_Bacilli; **o_Bacillales**						0.0041	5.7513	0.0276		
p_Firmicutes; c_Bacilli; o_Bacillales; **f_Bacillales incertae sedis**						0.0011				
p_Firmicutes; c_Bacilli; o_Bacillales; f_Bacillales incertae sedis; **g_Gemella**									22.437	1.1E-10
p_Firmicutes; c_Bacilli; o_Lactobacillales; **f_Aerococcaceae**						0.0011			21.698	4.4E-9
p_Firmicutes; c_Bacilli; o_Lactobacillales; f_Aerococcaceae; **g_Abiotrophia**									22.384	1.1E-10
p_Firmicutes; c_Bacilli; o_Lactobacillales; f_Aerococcaceae; **g_Abiotrophia; s_defectiva**						0.0043			23.83	9.0E-12
p_Firmicutes; c_Bacilli; o_Lactobacillales; **f_Enterococcaceae**						0.0124	12.004	0.0137	11.379	1.3E-6
p_Firmicutes; c_Bacilli; o_Lactobacillales; f_Enterococcaceae; **g_Enterococcus**							11.909	0.0341		
p_Firmicutes; c_Bacilli; o_Lactobacillales; f_Enterococcaceae; **g_Enterococcus; s_faecalis**									22.877	2.0E-13
p_Firmicutes; c_Clostridia; o_Clostridiales; f_Peptostreptococcaceae; **g_Peptostreptococcus**	1.6E-4									
p_Firmicutes; c_Erysipelotrichia; o_Erysipelotrichales; f_Erysipelotrichaceae; **g_Bulleidia**	0.0312									
p_Firmicutes; c_Negativicutes; o_Selenomonadales; f_Veillonellaceaea; **g_Megasphaera**	0.0169			23.501	5.9E-16					
p_Fusobacteria; c_Fusobacteriia; o_Fusobacteriales; f_Fusobacteriaceae; **g_Fusobacterium; s_periodonticum**	1.4E-5	9.9761	0.0053	9.6359	6.3E-5					
p_Proteobacteria; c_Gammaproteobacteria; **o_Enterobacteriales**	0.0019	7.7537	0.0024	10.752	3.4E-7					
p_Proteobacteria; c_Gammaproteobacteria; o_Enterobacteriales; **f_Enterobacteriaceae**	0.0021	6.998	0.0148	9.3791	1.5E-5					
p_Proteobacteria; c_Gammaproteobacteria; o_Enterobacteriales; f_Enterobacteriaceae; **g_Serratia**	1.6E-4									

This table showed the comparison of differential abundance of oral (in the middle columns) or fecal (on the right columns) samples between samples after 100 days from transplant and pre-HSCT time points. In square brackets, it was indicated the number of patients belonging to the groups compared in the analysis. The differential abundance statistical analysis for microbial taxa used different algorythms (zero-inflated Gaussian fit, EdgeR, or DESeq2). The complete taxonomy is indicated on the left column (*p_Phylum; c_Class; o_Order; f_Family; g_Genus; s_Species*). **FDR** (False Discovery Rate) indicates the statistical significance value after adjustment for multiple comparisons. The fold change

value indicated as logarithmic on base 2 (log2FC) represents how much is the increase/decrease of abundance of a particular taxa in the comparisons between the two group of samples. The FDR shown had values equal to or less than 0.05 and were considered statistically significant.

Table S4. Relative abundances of microbial taxa in oral or fecal samples comparing specimens developing oral mucositis (all grades) from the ones that never had such complication at engraftment and after 30 days from transplant.

	ORAL SWABS ORAL MUCOSITIS ALL GRADES OM+[10] *vs* OM- [5]					STOOLS SAMPLES ORAL MUCOSITIS ALL GRADES OM+ [8] *vs* OM-[5]				
**Taxonomy**	**zero-inflated Gaussian fit**	**EdgeR**		**DESeq2**		**zero-inflated Gaussian fit**	**EdgeR**		**DESeq2**	
	FDR	log2FC	FDR	log2FC	FDR	FDR	log2FC	FDR	log2FC	FDR
**Higher abundance in Oral Mucositis-positive samples at engraftment**										
p_Actinobacteria; c_Actinobacteria; ***o_Bifidobacteriales***							6.5911	0.0228		
p_Actinobacteria; c_Actinobacteria; o_Bifidobacteriales; ***f_Bifidobacteriaceae***							7.8998	0.0163		
p_Actinobacteria; c_Actinobacteria; o_Coriobacteriales; ***f_Coriobacteriaceae***	0.0320									
p_Actinobacteria; c_Actinobacteria; o_Coriobacteriales; f_Coriobacteriaceae; ***g_Atopobium***	0.0205									
p_Actinobacteria; c_Actinobacteria; o_Coriobacteriales; f_Coriobacteriaceae; ***g_Eggerthella***						0.0194	6.7501	0.0393	23.904	1.2E-12
p_Actinobacteria; c_Actinobacteria; o_Coriobacteriales; f_Coriobacteriaceae; ***g_Eggerthella; s_lenta***						0.0081	8.1478	0.0199	22.93	7.4E-12
p_Actinobacteria; ***c_Coriobacteriia***	0.0017									
p_Bacteroidetes; c_Bacteroidia; o_Bacteroidales; f_Bacteroidaceae; ***g_Bacteroides; s_dorei***						0.0080				
p_Bacteroidetes; c_Bacteroidia; o_Bacteroidales; f_Bacteroidaceae; ***g_Bacteroides; s_ovatus***						0.0062				
p_Bacteroidetes; c_Bacteroidia; o_Bacteroidales; f_Bacteroidaceae; ***g_Bacteroides; s_xylanisolvens***						0.0229	7.2536	0.0259		
p_Firmicutes; c_Bacilli; o_Lactobacillales; f_Enterococcaceae; ***g_Enterococcus; s_faecium***							6.3796	0.0281		
p_Firmicutes; c_Clostridia; o_Clostridiales; ***f_Clostridiales XI incertae sedis***	0.0388									
p_Firmicutes; c_Clostridia; o_Clostridiales; f_Lachnospiraceae; ***g_Blautia***						0.0211				
p_Firmicutes; c_Clostridia; o_Clostridiales; f_Lachnospiraceae; ***g_Blautia; s_producta***							5.3803	0.0280		
p_Firmicutes; c_Clostridia; o_Clostridiales; f_Lachnospiraceae; ***g_Roseburia***						0.0194				
p_Firmicutes; c_Clostridia; o_Clostridiales; f_Lachnospiraceae; ***g_Ruminococcus; s_gnavus***						0.0015	7.3397	0.0402		
p_Firmicutes; c_Clostridia; o_Clostridiales; f_Ruminococcaceae; ***g_Ruminococcus; s_sp.***						0.0297				
p_Firmicutes; c_Clostridia; o_Clostridiales; f_Ruminococcaceae; ***g_Faecalibacterium***						0.0311	6.4172	0.0459		
p_Firmicutes; c_Clostridia; o_Clostridiales; f_Ruminococcaceae; ***g_Faecalibacterium; s_prausnitzii***						0.0015	7.2946	0.0110		
p_Firmicutes; c_Clostridia; o_Clostridiales; f_Ruminococcaceae; ***g_Subdoligranulum***						0.0194				
p_Firmicutes; c_Clostridia; o_Clostridiales; f_Ruminococcaceae; ***g_Subdoligranulum; s_sp.***						0.0080	6.3371	0.0280		
p_Firmicutes; c_Clostridia; o_Clostridiales; f_unclassified Clostridiales; ***g_Flavonifractor; s_plautii***						0.0125				
p_Firmicutes; c_Erysipelotrichia; ***o_Erysipelotrichales***							6.1366	0.0345		
p_Firmicutes; c_Erysipelotrichia; o_Erysipelotrichales; ***f_Erysipelotrichaceae***							7.514	0.0255		
p_Firmicutes; c_Erysipelotrichia; o_Erysipelotrichales; f_Erysipelotrichaceae; ***g_Clostridium; s_cocleatum***						0.0038	7.5672	0.0249	24.931	8.1E-14
p_Firmicutes; c_Erysipelotrichia; o_Erysipelotrichales; f_Erysipelotrichaceae; ***g_Clostridium; s_innocuum***							6.2314	0.0259		
p_Firmicutes; c_Erysipelotrichia; o_Erysipelotrichales; f_Erysipelotrichaceae; ***g_Clostridium; s_spiroforme***						0.0174	8.4166	0.0249		
p_Firmicutes; c_Erysipelotrichia; o_Erysipelotrichales; f_Erysipelotrichaceae; ***g_[Eubacterium]***						0.0311				
p_Firmicutes; c_Erysipelotrichia; o_Erysipelotrichales; f_Erysipelotrichaceae; ***g_[Eubacterium]; s_dolichum***						0.0081	6.4618	0.0270		
p_Fusobacteria	0.0195									
p_Tenericutes		8.8447	0.0064	23.49	1.2E-12					
p_Tenericutes; ***c_Mollicutes***	0.0177	8.9135	0.0225	22.591	1.9E-11					
p_Tenericutes; c_Mollicutes; ***o_Mycoplasmatales***		12.608	0.0119	22.196	5.6E-11					
p_Tenericutes; c_Mollicutes; o_Mycoplasmatales; ***f_Mycoplasmataceae***		12.573	0.0334							
p_Tenericutes; c_Mollicutes; o_Mycoplasmatales; f_Mycoplasmataceae; ***g_Mycoplasma***	0.0481									
	**ORAL SWABS ORAL MUCOSITIS ALL GRADES OM+[7] *vs* OM- [3]**					**STOOLS ORAL MUCOSITIS ALL GRADES OM+ [7] *vs* OM-[3]**				
**Higher abundance in +30d Oral Mucositis-posivite**										
p_Bacteroidetes; c_Bacteroidia; o_Bacteroidales; f_Bacteroidaceae; ***g_Bacteroides; s_caccae***						0.0150				
p_Bacteroidetes; c_Bacteroidia; o_Bacteroidales; f_Bacteroidaceae; ***g_Bacteroides; s_ovatus***						0.0109			29.195	9.2E-17
p_Bacteroidetes; c_Bacteroidia; o_Bacteroidales; ***f_Prevotellaceae***	0.0188									

p_Bacteroidetes; c_Bacteroidia; o_Bacteroidales; f_Prevotellaceae; ***g_Prevotella***	0.0491									
p_Bacteroidetes; c_Bacteroidia; o_Bacteroidales; f_Prevotellaceae; ***g_Prevotella; s_oris***				25.0	3.3E-12					
p_Firmicutes; c_Bacilli; o_Lactobacillales; ***f_Aerococcaceae***									9.8823	0.0128
p_Firmicutes; c_Bacilli; o_Lactobacillales; f_Aerococcaceae; ***g_Abiotrophia***									10.351	0.0162
p_Firmicutes; c_Bacilli; o_Lactobacillales; f_Aerococcaceae; ***g_Abiotrophia; s_defectiva***									9.7971	0.0161
p_Firmicutes; c_Bacilli; o_Lactobacillales; f_Enterococcaceae; ***g_Enterococcus***						0.0105			8.6862	0.0307
p_Firmicutes; c_Bacilli; o_Lactobacillales; f_Enterococcaceae; ***g_Enterococcus; s_faecalis***									22.586	1.2E-10
p_Firmicutes; c_Bacilli; o_Lactobacillales; f_Streptococcaceae; ***g_Lactococcus***						0.0203			25.174	1.2E-12
p_Firmicutes; c_Bacilli; o_Lactobacillales; f_Streptococcaceae; ***g_Lactococcus; s_lactis***									23.05	4.9E-11
p_Firmicutes; c_Clostridia; o_Clostridiales; f_Clostridiaceae; ***g_Clostridium; s_aldenense***						0.0171			20.339	8.3E-9
p_Firmicutes; c_Clostridia; o_Clostridiales; f_Clostridiaceae; ***g_Clostridium; s_citroniae***									23.06	4.9E-11
p_Firmicutes; c_Clostridia; o_Clostridiales; f_Clostridiaceae; ***g_Clostridium; s_hathewayi***									25.375	5.4E-13
p_Firmicutes; c_Clostridia; o_Clostridiales; f_Clostridiaceae; ***g_Clostridium; s_lavalense***									22.392	1.7E-10
p_Firmicutes; c_Clostridia; o_Clostridiales; f_Lachnospiraceae; ***g_Catonella***	0.0065									
p_Firmicutes; c_Clostridia; o_Clostridiales; f_Lachnospiraceae; ***g_Catonella; s_morbi***				22.25	8.8E-10					
p_Firmicutes; c_Clostridia; o_Clostridiales; f_Lachnospiraceae; ***g_Lachnoclostridium***						0.0035			7.9187	0.0467
p_Firmicutes; c_Clostridia; o_Clostridiales; f_Lachnospiraceae; ***g_Lachnoclostridium; s_clostridioforme***						0.0365				
p_Firmicutes; c_Clostridia; o_Clostridiales; f_Lachnospiraceae; ***g_Lachnoclostridium; s_lavalense***									23.749	1.6E-11
p_Firmicutes; c_Clostridia; o_Clostridiales; f_Lachnospiraceae; ***g_Ruminococcus; s_gnavus***						0.0032			13.812	2.4E-5
p_Firmicutes; c_Clostridia; o_Clostridiales; ***f_Peptostreptococcaceae***	0.0300									
p_Firmicutes; c_Clostridia; o_Clostridiales; f_Peptostreptococcaceae; ***g_Peptostreptococcus***	0.0267			20.964	9.3E-9					
p_Firmicutes; c_Clostridia; o_Clostridiales; f_unclassified Clostridiales; ***g_Pseudoflavonifractor***									26.016	2.6E-13
p_Firmicutes; c_Negativicutes; o_Selenomonadales; f_Veillonellaceae; ***g_Selenomonas***				20.484	1.8E-8					
p_Firmicutes; c_Negativicutes; o_Selenomonadales; f_Veillonellaceae; ***g_Veillonella; s_caviae***									21.629	7.3E-10
p_Fusobacteria; c_Fusobacteriia; o_Fusobacteriales; f_Fusobacteriaceae; ***g_Fusobacterium; s_periodonticum***	0.0050									
p_Proteobacteria; c_Betaproteobacteria; o_Neisseriales; f_Neisseriaceae; ***g_Eikenella***	2.9E-4									
p_Proteobacteria; c_Epsilonproteobacteria; o_Campylobacterales; f_Campylobacteraceae; ***g_Campylobacter; s_curvus***				21.819	1.7E-9					
p_Proteobacteria; c_Gammaproteobacteria; o_Enterobacteriales; f_Enterobacteriaceae; ***g_Escherichia***									23.734	1.7E-11
p_Proteobacteria; c_Gammaproteobacteria; o_Enterobacteriales; f_Enterobacteriaceae; ***g_Serratia***	2.3E-4			23.448	1.3E-10					
**Higher abundance in samples that never developed Oral Mucositis**										
p_Actinobacteria							2.8799	0.0251	3.3736	0.0458
p_Actinobacteria; c_Actinobacteria; o_Actinomycetales; ***f_Micrococcaceae***		7.2379	0.0069				8.8045	0.0030		
p_Actinobacteria; c_Actinobacteria; o_Actinomycetales; f_Micrococcaceae; ***g_Rothia***							7.3508	0.0021		
p_Actinobacteria; c_Actinobacteria; o_Actinomycetales; f_Micrococcaceae; ***g_Rothia; s_aeria***		6.9626	0.0133	9.3756	0.0123					
p_Actinobacteria; c_Actinobacteria; o_Actinomycetales; f_Micrococcaceae; ***g_Rothia; s_denticariosa***		6.4381	0.0133	13.317	8.3E-5					
p_Actinobacteria; c_Actinobacteria; o_Actinomycetales; f_Micrococcaceae; ***g_Rothia; s_mucilaginosa***						0.0149	7.4151	0.0170		
p_Actinobacteria; c_Actinobacteria; o_Coriobacteriales; f_Coriobacteriaceae; ***g_Atopobium***							10.188	0.0014		
p_Actinobacteria; c_Actinobacteria; o_Coriobacteriales; f_Coriobacteriaceae; ***g_Atopobium; s_parvulum***							6.7019	0.0251		
p_Actinobacteria; c_Actinobacteria; o_Actinomycetales; f_Actinomycetaceae; ***g_Actinomyces***							5.374	0.0393		
p_Actinobacteria; c_Actinobacteria; o_Actinomycetales; f_Actinomycetaceae; ***g_Actinomyces; s_graevenitzii***		7.3065	0.0228							
p_Actinobacteria; c_Actinobacteria; o_Actinomycetales; f_Actinomycetaceae; ***g_Actinomyces; s_sp.***		5.821	0.0228	7.8075	0.0484		5.832	0.0199		
p_Actinobacteria; c_Actinobacteria; o_Actinomycetales; ***f_Corynebacteriaceae***	0.0048									
p_Actinobacteria; c_Actinobacteria; o_Actinomycetales; f_Corynebacteriaceae; ***g_Corynebacterium***	8.0E-4									
p_Actinobacteria; c_Actinobacteria; o_Actinomycetales; f_Micrococcaceae; ***g_Rothia***		6.8792	0.0259							
p_Actinobacteria; c_Actinobacteria; o_Actinomycetales; f_Micrococcaceae; ***g_Rothia; s_aeria***		6.4245	0.0364							
p_Actinobacteria; c_Actinobacteria; o_Actinomycetales; f_Micrococcaceae; ***g_Rothia; s_dentocariosa***		7.4444	0.0228							
p_Bacteroidetes; c_Bacteroidia; o_Bacteroidales; f_Bacteroidaceae; ***g_Bacteroides; s_fragilis***									29.313	7.4E-18
p_Bacteroidetes; c_Bacteroidia; o_Bacteroidales; f_Bacteroidaceae; ***g_Bacteroides; s_massiliensis***									28.164	9.2E-17
p_Bacteroidetes; c_Bacteroidia; o_Bacteroidales; f_Bacteroidaceae; ***g_Bacteroides; s_vulgatus***						1.3E-6	9.6794	0.0032	12.049	0.0011
p_Bacteroidetes; c_Bacteroidia; o_Bacteroidales; ***f_Prevotellaceae***									10.193	0.0104
p_Bacteroidetes; c_Flavobacteriia; o_Flavobacteriales; f_Flavobacteriaceae; ***g_Campnocytophaga; s_gingivalis***				8.814	0.0484					
p_Firmicutes; c_Bacilli; o_Bacillales; f_Bacillales incertae sedis; ***g_Gemella; s_sanguinis***				9.1973	0.0444		7.0731	0.0177	24.228	8.6E-13
p_Firmicutes; c_Bacilli; o_Lactobacillales; ***f_Carnobacteriaceae***							8.9802	0.0030	7.517	0.0104
p_Firmicutes; c_Bacilli; o_Lactobacillales; f_Carnobacteriaceae; ***g_Granulicatella***							5.7881	0.0139		
p_Firmicutes; c_Bacilli; o_Lactobacillales; f_Carnobacteriaceae; ***g_Granulicatella; s_adicens***							8.2407	0.0097		
p_Firmicutes; c_Bacilli; o_Lactobacillales; f_Carnobacteriaceae; ***g_Granulicatella; s_elegans***							4.6985	0.0199		
p_Firmicutes; c_Bacilli; o_Lactobacillales; f_Leuconostocaceae; ***g_Fructobacillus***							7.4336	0.0021		
p_Firmicutes; c_Bacilli; o_Lactobacillales; f_Leuconostocaceae; ***g_Fructobacillus; s_durionis***							6.1336	0.0083		

p_Firmicutes; c_Bacilli; o_Lactobacillales; f_Leuconostocaceae; ***g_Fructobacillus; s_fructosus***							5.1109	0.0142		
p_Firmicutes; c_Bacilli; o_Lactobacillales; f_Streptococcaceae; ***g_Streptococcus***	0.0087									
p_Firmicutes; c_Bacilli; o_Lactobacillales; f_Streptococcaceae; ***g_Streptococcus; s_australis***						0.0135	6.3179	0.0133		
p_Firmicutes; c_Bacilli; o_Lactobacillales; f_Streptococcaceae; ***g_Streptococcus; s_cristatus***				9.1541	0.0448					
p_Firmicutes; c_Bacilli; o_Lactobacillales; f_Streptococcaceae; ***g_Streptococcus; s_gordonii***				25.24	3.4E-13					
p_Firmicutes; c_Bacilli; o_Lactobacillales; f_Streptococcaceae; ***g_Streptococcus; s_lactarius***		6.6968	0.0228							
p_Firmicutes; c_Bacilli; o_Lactobacillales; f_Streptococcaceae; ***g_Streptococcus; s_mutans***		9.855	0.0076	30.0	2.1E-18					
p_Firmicutes; c_Bacilli; o_Lactobacillales; f_Streptococcaceae; ***g_Streptococcus; s_oralis***						0.0023	8.474	2.0E-5	8.3073	0.0098
p_Firmicutes; c_Bacilli; o_Lactobacillales; f_Streptococcaceae; ***g_Streptococcus; s_parasanguinis***							6.8144	4.8E-4	8.4047	0.0419
p_Firmicutes; c_Bacilli; o_Lactobacillales; f_Streptococcaceae; ***g_Streptococcus; s_peroris***						0.0297				
p_Firmicutes; c_Bacilli; o_Lactobacillales; f_Streptococcaceae; ***g_Streptococcus; s_salivarius***						5.6E-5	7.9938	6.3E-5	11.161	0.0035
p_Firmicutes; c_Clostridia; o_Clostridiales; ***f_Clostridiales Family XII_Incertae Sedis***							6.6949	0.0079		
p_Firmicutes; c_Clostridia; o_Clostridiales; f_Eubacteriaceae; ***g_Eubacterium***						0.0305			9.1064	0.0305
p_Firmicutes; c_Clostridia; o_Clostridiales; f_Lachnospiraceae; ***g_Lachnoanaerobaculum***							10.737	1.9E-4		
p_Firmicutes; c_Clostridia; o_Clostridiales; ***f_Oscillospiraceae***									24.789	3.5E-13
p_Firmicutes; c_Clostridia; o_Clostridiales; f_unclassified Clostridiales; ***g_Pseudoflavonifractor***							6.8419	0.0140		
p_Firmicutes; c_Clostridia; o_Clostridiales; f_Lachnospiraceae; ***g_Roseburia***						0.0012	9.7355	0.0021		
p_Firmicutes; c_Clostridia; o_Clostridiales; f_Lachnospiraceae; ***g_Roseburia; s_intestinalis***						4.8E-4	12.448	3.0E-4	30.0	2.1E-18
p_Firmicutes; c_Clostridia; o_Clostridiales; ***f_Ruminococcaceae***		5.449	0.0377	23.784	8.0E-12					
p_Firmicutes; c_Negativicutes; o_Selenomonadales; ***f_Acidaminococcacea***									26.375	1.4E-14
p_Firmicutes; c_Negativicutes; o_Selenomonadales; f_Acidaminococcacea; ***g_Phascolartobacterium***									27.662	7.4E-16
p_Firmicutes; c_Negativicutes; o_Selenomonadales; f_Veillonellaceae; ***g_Veillonella; s_dispar***							5.7076	0.0249	8.7972	0.0289
p_Fusobacteria							9.4661	8.4E-5	11.545	6.7E-4
p_Fusobacteria; ***c_Fusobacteriia***							8.2971	6.0E-4	9.7431	0.0155
p_Fusobacteria; c_Fusobacteriia; ***o_Fusobacteriales***							9.5969	3.6E-4	10.191	0.0083
p_Fusobacteria; c_Fusobacteriia; o_Fusobacteriales; ***f_Fusobacteriaceae***							8.0624	0.0079	23.221	9.1E-12
p_Fusobacteria; c_Fusobacteriia; o_Fusobacteriales; f_Fusobacteriaceae; ***g_Fusobacterium***							10.511	0.00120	9.3784	0.0269
p_Proteobacteria; c_Alphaproteobacteria; o_Rhizobiales; f_Hyphomicrobiaceae; ***g_Gemminger; s_formicilis***									26.227	9.7E-15
p_Proteobacteria; c_Deltaproteobacteria; ***o_Desulfovibrionales***							23.179	1.5E-11		
p_Proteobacteria; c_Deltaproteobacteria; o_Desulfovibrionales; f_Desulfovibrionaceae; ***g_Bilophila***									22.903	1.7E-11
p_Proteobacteria; c_Deltaproteobacteria; o_Desulfovibrionales; f_Desulfovibrionaceae; ***g_Bilophila; s_wadsworthia***									22.735	2.0E-11
p_Proteobacteria; ***c_Epsilonproteobacteria***							6.3346	0.0026	8.8419	0.0214
p_Proteobacteria; c_Epsilonproteobacteria; ***o_Campylobacterales***							5.9377	0.0039	8.8811	0.0242
p_Proteobacteria; c_Epsilonproteobacteria; o_Campylobacterales; ***f_Campylobacteraceae***							5.5684	0.0086	9.6948	0.0298
p_Proteobacteria; c_Epsilonproteobacteria; o_Campylobacterales; f_Campylobacteraceae; ***g_Campylobacter***						0.0311	5.8907	0.0340	9.4386	0.0269
p_Proteobacteria; c_Epsilonproteobacteria; o_Campylobacterales; f_Campylobacteraceae; ***g_Campylobacter; s_concisus***						2.7E-4	9.4494	6.3E-5	27.603	2.7E-17
p_Proteobacteria; c_Epsilonproteobacteria; o_Campylobacterales; f_Campylobacteraceae; ***g_Campylobacter; s_gracilis***						0.0492				
p_Proteobacteria; c_Gammaproteobacteria; o_Enterobacteriales; f_Enterobacteriaceae; ***g_Leclercia***							8.1943	0.0021		
p_Proteobacteria; c_Gammaproteobacteria; o_Enterobacteriales; f_Enterobacteriaceae; ***g_Leclercia; s_adecarboxylata***							7.9045	0.0083	8.6396	0.0302
p_Proteobacteria; c_Gammaproteobacteria; o_Enterobacteriales; f_Enterobacteriaceae; ***g_Klebsiella; s_oxytoca***							6.5351	0.0317	27.676	2.7E-16
p_Proteobacteria; c_Gammaproteobacteria; o_Enterobacteriales; f_Enterobacteriaceae; ***g_Klebsiella; s_sp.***									25.248	9.5E-14
p_Proteobacteria; c_Gammaproteobacteria; o_Enterobacteriales; f_Enterobacteriaceae; ***g_Providencia; s_stuartii***									22.841	1.7E-11
p_Proteobacteria; c_Gammaproteobacteria; ***o_Pasteurellales***						4.9E-4	9.0332	0.0025	6.9832	0.0279
p_Proteobacteria; c_Gammaproteobacteria; o_Pasteurellales; ***f_Pasteurellaceae***						1.3E-4	8.862	0.0067	7.1389	0.0158
p_Proteobacteria; c_Gammaproteobacteria; o_Pasteurellales; f_Pasteurellaceae; ***g_Haemophilus***						0.0012	9.3627	0.0021		
p_Proteobacteria; c_Gammaproteobacteria; o_Pasteurellales; f_Pasteurellaceae; ***g_Haemophilus; s_parainfluenzae***							5.0873	0.0249		
p_Proteobacteria; c_Gammaproteobacteria; o_Pasteurellales; f_Pasteurellaceae; ***g_Mannheimia***							6.4856	0.0437		
p_Proteobacteria; c_Gammaproteobacteria; o_Pasteurellales; f_Pasteurellaceae; ***g_Mannheimia; s_varigena***							6.3112	0.0498		

This table showed the comparison of differential abundance of oral (in the middle columns) or fecal (on the right columns) samples between patients that developed oral mucositis (all grades) from those who never reported it. The comparisons reported were at engraftment, or 30 days after transplant. In square brackets, it was indicated the number of patients belonging to the groups compared in the analysis. The differential abundance statistical analysis for microbial taxa used different algorythms (zero-inflated Gaussian fit, EdgeR, or DESeq2). The complete taxonomy is indicated on the left column (*p_Phylum; c_Class; o_Order; f_Family; g_Genus; s_Species*). **FDR** (False Discovery Rate) indicates

the statistical significance value after adjustment for multiple comparisons. The fold change value indicated as logarithmic on base 2 (log2FC) represents how much is the increase/decrease of abundance of a particular taxa in the comparisons between the two group of samples. The FDR shown had values equal to or less than 0.05 and were considered statistically significant.

Table S5. Relative abundances of microbial taxa in oral or fecal samples comparing specimens developing oral mucositis (grades >=2) from the ones that never had such complication at engraftment and after 30 days from transplant.

	ORAL SWABS ORAL MUCOSITES >=2 GRADES OM+[5] *vs* OM- [5]					STOOL SAMPLES ORAL MUCOSITES >=2 GRADES OM+[4] *vs* OM- [5]				
**Taxonomy**	**zero-inflated Gaussian fit**	**EdgeR**		**DESeq2**		**zero-inflated Gaussian fit**	**EdgeR**		**DESeq2**	
	FDR	log2FC	FDR	log2FC	FDR	FDR	log2FC	FDR	log2FC	FDR
**Higher abundance in Oral Mucositis-positive samples grades >=2 at engraftment**										
p_Actinobacteria; c_Actinobacteria; **o_Bifidobacteriales**							7.1343	0.0139		
p_Actinobacteria; c_Actinobacteria; o_Bifidobacteriales; **f_Bifidobacteriaceae**							7.8259	0.0139		
p_Actinobacteria; c_Actinobacteria; o_Bifidobacteriales; f_Bifidobacteriaceae; **g_Bifidobacterium; s_longum**						0.0239	4.5122	0.448		
p_Actinobacteria; c_Actinobacteria; o_Coriobacteriales; **f_Coriobacteriaceae**	0.0448									
p_Actinobacteria; c_Actinobacteria; o_Coriobacteriales; f_Coriobacteriaceae; **g_Atopobium**	0.0103									
p_Actinobacteria; c_Actinobacteria; o_Coriobacteriales; f_Coriobacteriaceae; **g_Atopobium; s_parvulum**		8.3056	0.0491							
p_Actinobacteria; c_Actinobacteria; o_Coriobacteriales; f_Coriobacteriaceae; **g_Eggerthella**						9.1E-5	8.1164	0.0350	27.746	8.5E-17
p_Actinobacteria; c_Actinobacteria; o_Coriobacteriales; f_Coriobacteriaceae; **g_Eggerthella; s_lenta**						6.0E-5	9.4115	0.0143	26.037	1.1E-14
p_Actinobacteria; **c_Coriobacteriia**	0.0304			5.0666	0.0495					
p_Bacteroidetes; c_Bacteroidia; o_Bacteroidales; f_Bacteroidaceae; **g_Bacteroides; s_ovatus**						1.4E-4	7.5418	0.0462		
p_Bacteroidetes; c_Bacteroidia; o_Bacteroidales; f_Porphyromonadaceae; **g_Odoribacter; s_splanchnicus**							5.7056	0.0255		
p_Bacteroidetes; c_Bacteroidia; o_Bacteroidales; f_Prevotellaceae; **g_Prevotella; s_oulorum**		8.9869	0.0491							
p_Firmicutes; c_Bacilli; o_Bacillales; f_Bacillales Incertae Sedis; **g_Gemella; s_morbillorum**	0.0320	10.058	0.0491							
p_Firmicutes; c_Bacilli; o_Lactobacillales; f_Enterococcaceae; **g_Enterococcus; s_faecium**						0.0352	7.4176	0.0150	11.005	0.0026
p_Firmicutes; c_Bacilli; o_Lactobacillales; f_Enterococcaceae; **g_Enterococcus; s_gallinarum**							4.3954	0.0438		
p_Firmicutes; c_Bacilli; o_Lactobacillales; f_Streptococcaceae; **g_Streptococcus; s_intermedius**						4.8E-4	6.2907	0.0438		
p_Firmicutes; c_Clostridia; o_Clostridiales; f_Clostridiaceae; **g_Clostridium; s_citroniae**						0.0161	7.469	0.0179		
p_Firmicutes; c_Clostridia; o_Clostridiales; f_Clostridiaceae; **g_Clostridium; s_tertium**							5.3303	0.0247	23.261	3.7E-12
p_Firmicutes; c_Clostridia; o_Clostridiales; **f_Clostridiales Family XI_Incertae Sedis**		8.0607	0.0484							
p_Firmicutes; c_Clostridia; o_Clostridiales; f_Lachnospiraceae; **g_Blautia; s_producta**							5.7554	0.0173		
p_Firmicutes; c_Clostridia; o_Clostridiales; f_Lachnospiraceae; **g_Ruminococcus; s_torques**						0.0250				
p_Firmicutes; c_Clostridia; o_Clostridiales; **f_Ruminococcaceae**	0.0131									
p_Firmicutes; c_Clostridia; o_Clostridiales; f_Ruminococcaceae; **g_Anaerotruncus; s_colihominis**							4.7947	0.0354		
p_Firmicutes; c_Clostridia; o_Clostridiales; f_Ruminococcaceae; **g_Faecalibacterium**						0.0089				
p_Firmicutes; c_Clostridia; o_Clostridiales; f_Ruminococcaceae; **g_Faecalibacterium; s_prausnitzii**						0.0012	8.554	0.0143		
p_Firmicutes; c_Clostridia; o_Clostridiales; f_Ruminococcaceae; **g_Ruminococcus**						0.0089				
p_Firmicutes; c_Clostridia; o_Clostridiales; f_Ruminococcaceae; **g_Ruminococcus; s_gnavus**						0.0033	8.1973	0.0186	7.1768	0.0161
p_Firmicutes; c_Clostridia; o_Clostridiales; f_Ruminococcaceae; **g_Ruminococcus; s_sp.**						0.0038	6.4368	0.0220		
p_Firmicutes; c_Clostridia; o_Clostridiales; f_Ruminococcaceae; **g_Subdoligranulum**						0.0089				
p_Firmicutes; c_Clostridia; o_Clostridiales; f_Ruminococcaceae; **g_Subdoligranulum; s_sp.**						0.0023	6.9532	0.0179		
p_Firmicutes; c_Clostridia; o_Clostridiales; f_unclassified Clostridiales; **g_Flavonifractor; s_plautii**						0.0250				
p_Firmicutes; **c_Erysipelotrichia**							5.466	0.0180	6.2668	0.0090
p_Firmicutes; c_Erysipelotrichia; **o_Erysipelotrichales**							6.4432	0.0201		
p_Firmicutes; c_Erysipelotrichia; **o_Erysipelotrichales; f_Erysipelotrichaceae**							7.4248	0.0134		
p_Firmicutes; c_Erysipelotrichia; o_Erysipelotrichales; f_Erysipelotrichaceae; **g_Clostridium; s_cocleatum**						2.3E-4	8.5318	0.0150	25.508	2.5E-14
p_Firmicutes; c_Erysipelotrichia; o_Erysipelotrichales; f_Erysipelotrichaceae; **g_Clostridium; s_innocuum**							5.1836	0.0383		
p_Firmicutes; c_Erysipelotrichia; o_Erysipelotrichales; f_Erysipelotrichaceae; **g_Clostridium; s_ramosum**						3.7E-4	8.1654	0.0150	25.312	3.4E-14
p_Firmicutes; c_Erysipelotrichia; o_Erysipelotrichales; f_Erysipelotrichaceae; **g_Clostridium; s_spiroforme**						0.0023	9.3331	0.0143	9.4331	0.0127
p_Firmicutes; c_Erysipelotrichia; o_Erysipelotrichales; f_Erysipelotrichaceae; **g_[Eubacterium]; s_dolichum**						0.0352	6.3136	0.0200		
p_Proteobacteria; c_Betaproteobacteria; o_Burkholderiales; f_Sutterellaceae; **g_Parasutterella; s_excrementihominis**						0.0239				
p_Proteobacteria; c_Epsilonproteobacteria; o_Campylobacterales; f_Campylobacteraceae; **g_Campylobacter; s_gracilis**		9.5411	0.0491							
p_Tenericutes; c_Mollicutes; o_Mycoplasmatales; f_Mycoplasmataceae; **g_Mycoplasma**				20.573	3.7E-10					

	ORAL SWABS<br>MUCOSITES >=2 GRADES<br>OM+[4] *vs* OM- [3]					FECAL SAMPLES<br>MUCOSITES >=2 GRADES<br>OM+[4] *vs* OM- [3]				
**Higher abundance in +30d Oral Mucositis-posivite grades >=2**										
p_Actinobacteria; c_Actinobacteria; o_Coriobacteriales; f_Coriobacteriaceae; ***g_Atopobium***	0.0394									
p_Bacteroidetes		0.8858	0.0391							
p_Bacteroidetes; c_Bacteroidia; o_Bacteroidales; f_Bacteroidaceae; ***g_Bacteroides; s_caccae***						0.0157				
p_Bacteroidetes; c_Bacteroidia; o_Bacteroidales; f_Bacteroidaceae; ***g_Bacteroides; s_ovatus***						0.0016			30.0	1.3E-14
p_Bacteroidetes; c_Bacteroidia; o_Bacteroidales; f_Bacteroidaceae; ***g_Bacteroides; s_xylanisolvens***									25.988	2.4E-11
p_Bacteroidetes; c_Bacteroidia; o_Bacteroidales; ***f_Prevotellaceae***	0.0110									
p_Bacteroidetes; c_Bacteroidia; o_Bacteroidales; f_Prevotellaceae; ***g_Prevotella; s_oris***				25.51	1.7E-10					
p_Firmicutes; c_Bacilli; o_Lactobacillales; ***f_Aerococcaceae***	0.0434									
p_Firmicutes; c_Bacilli; o_Lactobacillales; f_Enterococcaceae; ***g_Enterococcus***									9.3329	0.0122
p_Firmicutes; c_Bacilli; o_Lactobacillales; f_Enterococcaceae; ***g_Enterococcus; s_faecalis***									9.1495	0.0444
p_Firmicutes; c_Clostridia; o_Clostridiales; ***f_Clostridiales family XI incertae sedis***	0.0434									
p_Firmicutes; c_Clostridia; o_Clostridiales; f_Clostridiaceae; ***g_Clostridium; s_aldenense***						0.0452			24.809	1.9E-10
p_Firmicutes; c_Clostridia; o_Clostridiales; f_Clostridiaceae; ***g_Clostridium; s_citroniae***									10.27	0.0198
p_Firmicutes; c_Clostridia; o_Clostridiales; f_Clostridiaceae; ***g_Clostridium; s_hathewayi***						0.0042			26.038	2.4E-11
p_Firmicutes; c_Clostridia; o_Clostridiales; f_Clostridiaceae; ***g_Clostridium; s_lavalense***						0.0347			27.404	2.3E-12
p_Firmicutes; c_Clostridia; o_Clostridiales; f_Lachnospiraceae; ***g_Catonella; s_morbi***	0.0196									
p_Firmicutes; c_Clostridia; o_Clostridiales; f_Lachnospiraceae; ***g_Lachnoanaerobaculum; s_umeaense***	0.0429									
p_Firmicutes; c_Clostridia; o_Clostridiales; f_Lachnospiraceae; ***g_Lachnoclostridium***									8.8993	0.0122
p_Firmicutes; c_Clostridia; o_Clostridiales; f_Lachnospiraceae; ***g_Lachnoclostridium; s_lavalense***						0.0396			24.641	2.3E-10
p_Firmicutes; c_Clostridia; o_Clostridiales; f_Lachnospiraceae; ***g_Oribacterium***	0.0394									
p_Firmicutes; c_Clostridia; o_Clostridiales; f_Lachnospiraceae; ***g_Ruminococcus; s_gnavus***						0.0016			14.29	6.1E-7
p_Firmicutes; c_Clostridia; o_Clostridiales; ***f_Peptostreptococcaceae***	0.0110			8.931	0.0414					
p_Firmicutes; c_Clostridia; o_Clostridiales; f_unclassified Clostridiales; ***g_Pseudoflavonifractor***									26.774	1.2E-11
p_Proteobacteria; c_Alphaproteobacteria; o_Rhizobiales; f_Hyphomicrobiaceae; ***g_Gemminger; s_formicilis***						0.0396				
p_Proteobacteria; c_Epsilonproteobacteria; o_Campylobacterales; f_Campylobacteraceae; ***g_Campylobacter; s_curvus***	0.0429									
**Higher abundance in samples that never developed Oral Mucositis**										
p_Actinobacteria; c_Actinobacteria; o_Actinomycetales; ***f_Corynebacteriaceae***				8.1902	0.0418				24.178	1.0E-12
p_Actinobacteria; c_Actinobacteria; o_Actinomycetales; f_Corynebacteriaceae; ***g_Corynebacterium***	0.0394									
p_Actinobacteria; c_Actinobacteria; o_Actinomycetales; ***f_Micrococcaceae***							11.64	0.0324		
p_Actinobacteria; c_Actinobacteria; o_Actinomycetales; ***f_Micrococcaceae; g_Rothia***							10.951	0.0412		
p_Actinobacteria; c_Actinobacteria; o_Actinomycetales; ***f_Micrococcaceae; g_Rothia; s_aeria***				26.98	1.3E-12					
p_Actinobacteria; c_Actinobacteria; ***o_Coriobacteriales***							6.2371	0.0494		
p_Actinobacteria; c_Actinobacteria; ***o_Coriobacteriales; f_Coriobacteriaceae***							6.6238	0.0409		
p_Bacteroidetes; c_Bacteroidia; o_Bacteroidales; f_Bacteroidaceae; ***g_Bacteroides; s_vulgatus***						0.0051			10.208	0.0048
p_Firmicutes; c_Bacilli; o_Lactobacillales; f_Aerococcaceae; ***g_Abiotrophia; s_defectiva***						0.0228				
p_Firmicutes; c_Bacilli; o_Lactobacillales; ***f_Carnobacteriaceae***							11.008	0.0324		
p_Firmicutes; c_Bacilli; o_Lactobacillales; f_Carnobacteriaceae; ***g_Granulicatella***							11.326	0.0412		
p_Firmicutes; c_Bacilli; o_Lactobacillales; f_Carnobacteriaceae; ***g_Granulicatella; s_elegans***	0.0429									
p_Firmicutes; c_Bacilli; o_Lactobacillales; f_Streptococcaceae; ***g_Streptococcus; s_infantis***							7.5586	0.0200	8.4964	0.0161
p_Firmicutes; c_Bacilli; o_Lactobacillales; f_Streptococcaceae; ***g_Streptococcus; s_oralis***							6.3419	0.0404	6.8374	0.0210
p_Firmicutes; c_Bacilli; o_Lactobacillales; f_Streptococcaceae; ***g_Streptococcus; s_pneumoniae***						0.0156	6.1692	0.0255	8.0452	0.0485
p_Firmicutes; c_Bacilli; o_Lactobacillales; f_Streptococcaceae; ***g_Streptococcus; s_salivarius***						0.0011	8.1837	0.0179	11.319	0.0024
p_Firmicutes; c_Clostridia; o_Clostridiales; ***f_Clostridiales Family XII_Incertae Sedis***							9.2216	0.0422		
p_Firmicutes; c_Clostridia; o_Clostridiales; f_Clostridiales Family XIII_Incertae Sedis; ***g_Mogibacterium***									23.046	8.4E-12
p_Firmicutes; c_Clostridia; o_Clostridiales; f_Lachnospiraceae; ***g_Lachnoanaerobaculum; s_orale***							8.9287	0.0247	23.035	8.3E-12
p_Firmicutes; c_Clostridia; o_Clostridiales; f_Lachnospiraceae; ***g_Lachnoanaerobaculum; s_sp.***							6.9764	0.0329	22.2	4.9E-11
p_Firmicutes; c_Clostridia; o_Clostridiales; f_Lachnospiraceae; ***g_Roseburia***							9.9051	0.0439		
p_Firmicutes; c_Clostridia; o_Clostridiales; f_Lachnospiraceae; ***g_Roseburia; s_intestinalis***						0.0354			29.989	1.1E-14
p_Firmicutes; ***c_Negativicutes***						0.0062	12.632	6.0E-5	13.65	1.9E-8
p_Firmicutes; c_Negativicutes; ***o_Selenomonadales***						0.0134	11.695	0.0016	14.763	1.8E-11
p_Firmicutes; c_Negativicutes; o_Selenomonadales; ***f_Veillonellaceae***							11.193	0.0109	14.777	6.9E-8
p_Firmicutes; c_Negativicutes; o_Selenomonadales; f_Veillonellaceae; ***g_Megasphaere***						0.0453			26.877	9.2E-16
p_Firmicutes; c_Negativicutes; o_Selenomonadales; f_Veillonellaceae; ***g_Veillonella***						9.1E-5	12.753	0.0325	29.073	3.9E-23

p_Firmicutes; c_Negativicutes; o_Selenomonadales; f_Veillonellaceae; ***g_Veillonella; s_alcalescens***						4.9E-4	9.4603	0.0173	11.601	3.3E-4
p_Firmicutes; c_Negativicutes; o_Selenomonadales; f_Veillonellaceae; ***g_Veillonella; s_atypica***						0.0473	9.0337	0.0247	25.962	1.9E-14
p_Firmicutes; c_Negativicutes; o_Selenomonadales; f_Veillonellaceae; ***g_Veillonella; s_denticariosi***							8.318	0.0247	25.07	9.9E-14
p_Firmicutes; c_Negativicutes; o_Selenomonadales; f_Veillonellaceae; ***g_Veillonella; s_dispar***						0.0415	10.223	0.0217	27.043	4.6E-15
p_Firmicutes; c_Negativicutes; o_Selenomonadales; f_Veillonellaceae; ***g_Veillonella; s_parvula***						0.0161	6.3204	0.0255	8.2906	0.0412
p_Firmicutes; c_Negativicutes; o_Selenomonadales; f_Veillonellaceae; ***g_Veillonella; s_rogosae***						0.0033	8.9057	0.0173	10.92	0.0012
p_Firmicutes; c_Negativicutes; o_Selenomonadales; f_Veillonellaceae; ***g_Veillonella; s_tobetsuensis***						0.0415	8.138	0.0220	10.528	0.0058
p_Fusobacteria; c_Fusobacteriia; ***o_Fusobacteriales***							8.3241	0.0286		
p_Fusobacteria; c_Fusobacteriia; o_Fusobacteriales; f_Leptotrichiaceae; ***g_Leptotrichia***	0.0103									
p_Proteobacteria; ***c_Epsilonproteobacteria***						0.0062	10.193	0.0180	25.616	2.4E-14
p_Proteobacteria; c_Epsilonproteobacteria; ***o_Campylobacterales***						0.0134	9.0013	0.0139	26.436	3.5E-15
p_Proteobacteria; c_Epsilonproteobacteria; o_Campylobacterales; ***f_Campylobacteraceae***							8.5853	0.0211	26.785	7.4E-16
p_Proteobacteria; c_Epsilonproteobacteria; o_Campylobacterales; f_Campylobacteraceae; ***g_Campylobacter***						0.0019			27.136	6.2E-16
p_Proteobacteria; c_Epsilonproteobacteria; o_Campylobacterales; f_Campylobacteraceae; ***g_Campylobacter; s_concisus***						0.0019	9.5707	0.0200	26.404	1.1E-14
p_Proteobacteria; c_Epsilonproteobacteria; o_Campylobacterales; f_Campylobacteraceae; ***g_Campylobacter; s_gracilis***							6.2689	0.0354		
p_Proteobacteria; ***c_Gammaproteobacteria***	0.0304					0.0182				
p_Proteobacteria; c_Gammaproteobacteria; ***o_Pasteurellales***							8.0171	0.0201		
p_Proteobacteria; c_Gammaproteobacteria; o_Pasteurellales; f_Pasteurellaceae; ***g_Haemophilus***							10.913	0.0412	22.666	1.6E-11
p_Proteobacteria; c_Gammaproteobacteria; o_Pasteurellales; f_Pasteurellaceae; ***g_Haemophilus; s_parainfluenzae***							8.6652	0.0247	23.235	5.9E-12
p_Proteobacteria; c_Gammaproteobacteria; o_Pasteurellales; f_Pasteurellaceae; ***g_Mannheimia***	0.0033									

This table showed the comparison of differential abundance of oral (in the middle columns) or fecal (on the right columns) samples between patients that developed oral mucositis (grade>=2) from those who never reported it. The comparisons reported were at engraftment, or 30 days after transplant. In square brackets, it was indicated the number of patients belonging to the groups compared in the analysis. The differential abundance statistical analysis for microbial taxa used different algorythms (zero-inflated Gaussian fit, EdgeR, or DESeq2). The complete taxonomy is indicated on the left column (*p_Phylum; c_Class; o_Order; f_Family; g_Genus; s_Species*). **FDR** (False Discovery Rate) indicates the statistical significance value after adjustment for multiple comparisons. The fold change value indicated as logarithmic on base 2 (log2FC) represents how much is the increase/decrease of abundance of a particular taxa in the comparisons between the two group of samples. The FDR shown had values equal to or less than 0.05 and were considered statistically significant.

Table S6. Relative abundances of microbial taxa in fecal samples comparing patients with acute cutaneous GvHD from the ones that never developed it. The analysis was performed before the transplant, at the engraftment, and 30 days from HSCT.

	STOOLS<br>acute cutaneous GvHD<br>aGvHD-Positive [6] *vs* aGvHD-Negative [7]									
**Taxonomy**	**zero-inflated Gaussian fit**	**EdgeR**		**DESeq2**		**zero-inflated Gaussian fit**	**EdgeR**		**DESeq2**	
	FDR	log2FC	FDR	log2FC	FDR	FDR	log2FC	FDR	log2FC	FDR
	**Higher abundance in cutaneous aGvHD-positive samples pre HSCT-transplant**					**Higher abundance in cutaneous aGvHD-negative samples pre HSCT-transplant**				
p_Actinobacteria; c_Actinobacteria; o_Bifidobacteriales; f_Bifidobacteriaceae; **g_Bifidobacterium; s_longum**	0.0225									
p_Bacteroidetes; c_Bacteroidia; o_Bacteroidales; f_Bacteroidaceae; **g_Bacteroides; s_finegoldi**						0.0188				
p_Bacteroidetes; c_Bacteroidia; o_Bacteroidales; f_Bacteroidaceae; **g_Bacteroides; s_fragilis**				9.5756	0.0203					
p_Bacteroidetes; c_Bacteroidia; o_Bacteroidales; f_Bacteroidaceae; **g_Bacteroides; s_stercoris**						0.0188				
p_Bacteroidetes; c_Bacteroidia; o_Bacteroidales; f_Rikenellaceae; **g_Alistipes; s_putredinis**						0.0011	7.9544	0.0414	26.526	1.3E-16
p_Bacteroidetes; c_Bacteroidia; o_Bacteroidales; f_ Prevotellaceae; **g_Prevotella**							10.761	0.0370		
**p_Firmicutes**							2.7292	0.0449		
p_Firmicutes; c_Bacilli; o_Lactobacillales; f_Lactobacillaceae; **g_Lactobacillus; s_rogosae**				21.505	1.9E-11					
p_Firmicutes; c_Bacilli; o_Lactobacillales; f_Streptococcaceae; **g_Lactococcus; s_lactis**				25.384	1.2E-15					
p_Firmicutes; c_Clostridia; o_Clostridiales; f_Clostridiaceae; **g_Clostridium; s_saccharoperbutylacetonicum**	0.0188	8.696	0.0395	9.6625	0.0206					
p_Firmicutes; c_Clostridia; o_Clostridiales; f_Lachnospiraceae; **g_Anaerostipes**							7.1387	0.0370		
p_Firmicutes; c_Clostridia; o_Clostridiales; f_Lachnospiraceae; **g_Roseburia; s_intestinalis**				22.575	1.7E-12					
p_Firmicutes; c_Clostridia; o_Clostridiales; f_Ruminococcaceae; **g_Ruminococcus; s_bromii**	0.0010									
p_Firmicutes; c_Clostridia; o_Clostridiales; f_Ruminococcaceae; **g_Ruminococcus; s_gnavus**	0.0255									
p_Firmicutes; c_Negativicutes; o_Selenomonadales; f_Veillonellaceae; **g_Veillonella; s_alcalescens**	0.0198									
p_Firmicutes; c_Negativicutes; o_Selenomonadales; f_Veillonellaceae; **g_Veillonella; s_rogosae**	0.0255									

	STOOLS<br>acute cutaneous GvHD<br>aGvHD-Positive [5] *vs* aGvHD-Negative [7]									
**Taxonomy**	**zero-inflated Gaussian fit**	**EdgeR**		**DESeq2**		**zero-inflated Gaussian fit**	**EdgeR**		**DESeq2**	
	FDR	log2FC	FDR	log2FC	FDR	FDR	log2FC	FDR	log2FC	FDR
	**Higher abundance in cutaneous aGvHD-positive samples at engraftment**					**Higher abundance in cutaneous aGvHD-negative samples at engraftment**				
p_Actinobacteria; c_Actinobacteria; **o_Bifidobacteriales**		4.3691	0.0342							
p_Actinobacteria; c_Actinobacteria; o_Coriobacteriales; f_Coriobacteriaceae; **g_Eggerthella**	0.0428	5.7107	0.0153							
p_Actinobacteria; c_Actinobacteria; o_Coriobacteriales; f_Coriobacteriaceae; **g_Eggerthella; s_lenta**	0.0117	7.7489	0.0061							
p_Bacteroidetes; c_Bacteroidia; o_Bacteroidales; f_Bacteroidaceae; **g_Bacteroides; s_thetaiotaomicron**	0.0073	5.3563	0.0331							
p_Bacteroidetes; c_Bacteroidia; o_Bacteroidales; f_Bacteroidaceae; **g_Bacteroides; s_vulgatus**	4.8E-4	10.616	0.0132	13.629	1.1E-4					
p_Bacteroidetes; c_Bacteroidia; o_Bacteroidales; f_Bacteroidaceae; **g_Bacteroides; s_xylanisolvens**	0.0094	7.8764	0.0061							
p_Bacteroidetes; c_Bacteroidia; o_Bacteroidales; f_Rikenellaceae; **g_Alistipes**	0.0443									
p_Bacteroidetes; c_Bacteroidia; o_Bacteroidales; f_Rikenellaceae; **g_Alistipes; s_onderdonkii**	1.3E-4	7.0629	0.0367							
**p_Firmicutes**							6.5293	0.0130		
p_Firmicutes; **c_Bacilli**							7.0344	0.0396		
p_Firmicutes; c_Bacilli; **o_Lactobacillales**							6.3721	0.0342		
p_Firmicutes; c_Bacilli; o_Lactobacillales; f_Streptococcaceae; **g_Lactococcus; s_lactis**				25.384	1.2E-15					
p_Firmicutes; c_Clostridia; o_Clostridiales; f_Clostridiaceae; **g_Clostridium; s_innocuum**	0.0432	7.3198	0.0059							
p_Firmicutes; c_Clostridia; o_Clostridiales; f_Eubacteriaceae; **g_Eubacterium; s_dolichum**	0.0028	7.5566	0.0059							

p_Firmicutes; c_Clostridia; o_Clostridiales; f_Lachnospiraceae; **g_Lachnoclostridium; s_clostridioforme**		3.8026	0.0367							
p_Firmicutes; c_Clostridia; o_Clostridiales; f_Ruminococcaceae; **g_Anaerotruncus; s_colihominis**		4.0257	0.0217							
p_Firmicutes; c_Clostridia; o_Clostridiales; f_Ruminococcaceae; **g_Ruminococcus; s_sp.**	0.0094	6.4195	0.0085							
p_Firmicutes; c_Clostridia; o_Clostridiales; f_Ruminococcaceae; **g_Ruminococcus; s_torques**	0.0276	5.8732	0.0367							
p_Firmicutes; c_Erysipelotrichia; o_Erysipelotrichales; f_Erysipelotrichaceae; **g_[Eubacterium]**	0.0443	5.6514	0.0153							
p_Firmicutes; **c_Negativicutes**							8.2279	0.0396		
p_Firmicutes; c_Negativicutes; **o_Selenomonadales**							7.9248	0.342		
p_Firmicutes; c_Negativicutes; o_Selenomonadales; f_Veillonellaceae; **g_Veillonella**						0.0443				
p_Firmicutes; c_Negativicutes; o_Selenomonadales; f_Veillonellaceae; **g_Veillonella; s_alcalescens**						5.0E-4				
p_Firmicutes; c_Negativicutes; o_Selenomonadales; f_Veillonellaceae; **g_Veillonella; s_parvula**						4.8E-4	7.4506	0.0217	25.417	7.3E-15
p_Firmicutes; c_Negativicutes; o_Selenomonadales; f_Veillonellaceae; **g_Veillonella; s_rogosae**						4.8E-4				
p_Proteobacteria; c_Deltaproteobacteria; o_Desulfovibrionales; f_Desulfovibrionaceae; **g_Bilophila**		6.2882	0.0447							
p_Proteobacteria; c_Gammaproteobacteria; o_Enterobacteriales; f_Enterobacteriaceae; **g_Cronobacter; s_turicensis**				9.6924	0.0265					
p_Proteobacteria; c_Gammaproteobacteria; o_Enterobacteriales; f_Enterobacteriaceae; **g_Klebsiella; s_pneumoniae**	0.0145									
p_Proteobacteria; c_Gammaproteobacteria; o_Enterobacteriales; f_Enterobacteriaceae; **g_Morganella; s_morganii**		4.807	0.0217							
p_Proteobacteria; c_Gammaproteobacteria; o_Enterobacteriales; f_Enterobacteriaceae; **g_Providencia**	0.0443	5.0666	0.0243							
p_Proteobacteria; c_Gammaproteobacteria; o_Enterobacteriales; f_Enterobacteriaceae; **g_Yersinia**		4.7021	0.0243							

	**STOOLS**<br>**acute cutaneous GvHD**<br>**aGvHD-Positive [4] *vs* aGvHD-Negative [5]**									
**Taxonomy**	**zero-inflated Gaussian fit**	**EdgeR**		**DESeq2**		**zero-inflated Gaussian fit**	**EdgeR**		**DESeq2**	
	FDR	log2FC	FDR	log2FC	FDR	FDR	log2FC	FDR	log2FC	FDR
	**Higher abundance in cutaneous aGvHD-positive samples after 30 days from HSCT**					**Higher abundance in cutaneous aGvHD-negative samples after 30 days from HSCT**				
p_Actinobacteria; c_Actinobacteria; o_Bifidobacteriales; f_Bifidobacteriaceae; **g_Bifidobacterium; s_longum**	0.0047	6.4298	0.0296	11.453	0.0013					
p_Actinobacteria; c_Actinobacteria; o_Actinomycetales; **f_Micrococcaceae**							8.5332	0.0490		
p_Bacteroidetes; c_Bacteroidia; o_Bacteroidales; f_Bacteroidaceae; **g_Bacteroides; s_caccae**	1.1E-5	13.274	0.0161	30.0	5.4E-19					
p_Bacteroidetes; c_Bacteroidia; o_Bacteroidales; f_Bacteroidaceae; **g_Bacteroides; s_fragilis**	0.0115									
p_Bacteroidetes; c_Bacteroidia; o_Bacteroidales; f_Bacteroidaceae; **g_Bacteroides; s_massiliensis**	1.1E-6	9.8137	0.0161	29.213	1.8E-18					
p_Bacteroidetes; c_Bacteroidia; o_Bacteroidales; f_Bacteroidaceae; **g_Bacteroides; s_vulgatus**	0.0018	8.3649	0.0161	13.038	1.5E-4					
p_Bacteroidetes; c_Bacteroidia; o_Bacteroidales; **f_Porphyromonadaceae**		5.7921	0.0245							
p_Bacteroidetes; c_Bacteroidia; o_Bacteroidales; f_Porphyromonadaceae; **g_Parabacteroides**		7.0381	0.0353	8.1603	0.0495					
p_Bacteroidetes; c_Bacteroidia; o_Bacteroidales; f_Porphyromonadaceae; **g_Parabacteroides; s_distasonis**	0.0025	6.2414	0.0494							
p_Bacteroidetes; c_Bacteroidia; o_Bacteroidales; **f_Prevotellaceae**	0.0323	7.5055	0.0196	27.637	5.1E-17					
p_Bacteroidetes; c_Bacteroidia; o_Bacteroidales; f_Prevotellaceae; **g_Prevotella**	0.0035	11.113	0.0198	29.972	3.2E-19					
p_Firmicutes; c_Bacilli; **o_Bacillales**						0.0314				
p_Firmicutes; c_Bacilli; o_Bacillales; **f_Bacillales incertae sedis**							9.0221	0.0359		
p_Firmicutes; c_Bacilli; o_Bacillales; f_Bacillales incertae sedis; **g_Gemella; s_morbillorum**							6.4323	0.0410		
p_Firmicutes; c_Bacilli; o_Lactobacillales; **f_Carnobacteriaceae**						0.0281	9.3131	0.0359		
p_Firmicutes; c_Bacilli; o_Lactobacillales;f_Leuconostocaceae; **g_Weissella**	0.0183	6.2806	0.0353	27.512	9.0E-17					
p_Firmicutes; c_Bacilli; o_Lactobacillales;f_Leuconostocaceae; **g_Weissella; s_cibaria**	0.0268									
p_Firmicutes; c_Bacilli; o_Lactobacillales;f_Leuconostocaceae; **g_Weissella; s_confusa**	0.0268	5.6127	0.0410	25.595	1.4E-14					
p_Firmicutes; c_Clostridia; o_Clostridiales; f_Clostridiaceae; **g_Clostridium; s_hathewayi**	0.0428									
p_Firmicutes; c_Clostridia; o_Clostridiales; f_Clostridiaceae; **g_Clostridium; s_lavalense**	0.0019	9.3067	0.0161	27.81	1.6E-17					
p_Firmicutes; c_Clostridia; o_Clostridiales; f_Clostridiaceae; **g_Clostridium; s_sp.**	0.0268									
p_Firmicutes; c_Clostridia; o_Clostridiales; f_Eubacteriaceae; **g_Eubacterium**	0.0327			28.396	3.9E-18					
p_Firmicutes; c_Clostridia; o_Clostridiales; f_Lachnospiraceae; **g_Blautia; s_coccoides**									22.067	6.3E-11
p_Firmicutes; c_Clostridia; o_Clostridiales; f_Lachnospiraceae; **g_Blautia; s_wexlerae**	0.0152	5.4903	0.0410	25.399	1.9E-14					
p_Firmicutes; c_Clostridia; o_Clostridiales; f_Lachnospiraceae; **g_Lachnoclostridium; s_clostridioforme**	0.0207	8.266	0.0320	26.595	1.3E-15					
p_Firmicutes; c_Clostridia; o_Clostridiales; f_Ruminococcaceae; **g_Faecalibacterium; s_prausnitzii**	0.0115									
p_Firmicutes; c_Erysipelotrichia; o_Erysipelotrichales; Erysipelotrichaceae; **g_[Eubacterium]**		9.4861	0.0198							
p_Firmicutes; c_Negativicutes; o_Selenomonadales; **f_Acidaminococcaceae**	0.0378	7.0215	0.0196	27.777	5.1E-17					
p_Fusobacteria; c_Fusobacteriia; o_Fusobacteriales; **f_Fusobacteriaceae**							6.9335	0.0359	22.061	5.4E-11

p_Proteobacteria; **c_Alphaproteobacteria**	0.0332			7.686	0.0430					
p_Proteobacteria; c_Alphaproteobacteria; **o_Rhizobiales**	0.0021	8.7374	0.0129	29.623	3.0E-19					
p_Proteobacteria; c_Alphaproteobacteria; o_Rhizobiales; **f_Hyphomicrobiaceae**	0.0281	8.0368	0.0196	28.926	4.4E-18					
p_Proteobacteria; c_Alphaproteobacteria; o_Rhizobiales; **f_Hyphomicrobiaceae; g_Gemminger; s_formicilis**	5.0E-5	9.9142	0.0161	29.75	5.7E-19					
p_Proteobacteria; **c_Betaproteobacteria**	0.0450	6.2335	0.0438							
p_Proteobacteria; c_Betaproteobacteria; **o_Burkholderiales**		5.2019	0.0366							
p_Proteobacteria; c_Betaproteobacteria; **o_Burkholderiales; f_Sutterellaceae**		4.8734	0.0359							
p_Proteobacteria; c_Betaproteobacteria; o_Burkholderiales; f_Sutterellaceae; **g_Parasutterella**	0.0037	7.9613	0.0198	29.505	6.4E-19					
p_Proteobacteria; **c_Deltaproteobacteria**	0.0332	7.037	0.0371	25.489	1.9E-14					
p_Proteobacteria; c_Deltaproteobacteria; **o_Desulfovibrionales**	0.0314	5.6968	0.0366	26.442	1.1E-15					
p_Proteobacteria; c_Deltaproteobacteria; **o_Desulfovibrionales; f_Desulfovibrionaceae**		5.0192	0.0359	25.952	3.7E-15					
p_Proteobacteria; c_Deltaproteobacteria; o_Desulfovibrionales; f_Desulfovibrionaceae; **g_Bilophila; s_wadsworthia**	0.0047	6.7614	0.0320	26.7	1.18E-15					
p_Proteobacteria; c_Gammaproteobacteria; o_Enterobacteriales; f_Enterobacteriaceae; **g_Citrobacter; s_werkmanii**	0.0196									
p_Proteobacteria; c_Gammaproteobacteria; o_Enterobacteriales; f_Enterobacteriaceae; **g_Enterobacter**						0.0327	7.1412	0.0374		
p_Proteobacteria; c_Gammaproteobacteria; o_Enterobacteriales; f_Enterobacteriaceae; **g_Enterobacter; s_sacchari**						0.0268				
p_Proteobacteria; c_Gammaproteobacteria; o_Enterobacteriales; f_Enterobacteriaceae; **g_Escherichia**						0.0451	6.3608	0.0374	23.075	7.2E-12
p_Proteobacteria; c_Gammaproteobacteria; o_Enterobacteriales; f_Enterobacteriaceae; **g_Escherichia; s_coli**							6.4765	0.0482	23.352	3.9E-12
p_Proteobacteria; c_Gammaproteobacteria; o_Enterobacteriales; f_Enterobacteriaceae; **g_Escherichia/Shigella**							6.1816	0.0374	23.013	7.2E-12
p_Proteobacteria; c_Gammaproteobacteria; o_Enterobacteriales; f_Enterobacteriaceae; **g_Klebsiella; s_oxytoca**							9.19	0.0410	25.832	1.4E-14
p_Proteobacteria; c_Gammaproteobacteria; o_Enterobacteriales; f_Enterobacteriaceae; **g_Leclercia**						0.0451	8.3258	0.0370	24.259	5.06E-13
p_Proteobacteria; c_Gammaproteobacteria; o_Enterobacteriales; f_Enterobacteriaceae; **g_Leclercia; s_adecarboxylata**							8.763	0.0410	25.408	3.2E-14
p_Proteobacteria; c_Gammaproteobacteria; o_Enterobacteriales; f_Enterobacteriaceae; **g_Salmonella**							4.7185	0.0476		

This table showed the comparison of differential abundance of fecal samples between patients that developed acute cutaneous GvHD (on the middle columns) from those who never reported it (on the right columns) at the different time points (pre-HSCT, engraftment and 30 days after transplant). In square brackets, it was indicated the number of patients belonging to the groups compared in the analysis. The differential abundance statistical analysis for microbial taxa used different algorythms (zero-inflated Gaussian fit, EdgeR, or DESeq2). The complete taxonomy is indicated on the left column (*p_Phylum; c_Class; o_Order; f_Family; g_Genus; s_Species*). **FDR** (False Discovery Rate) indicates the statistical significance value after adjustment for multiple comparisons. The fold change value indicated as logarithmic on base 2 (log2FC) represents how much is the increase/decrease of abundance of a particular taxa in the comparisons between the two group of samples. The FDR shown had values equal to or less than 0.05 and were considered statistically significant.
